# Regulation of keratin network dynamics by the mechanical properties of the environment in migrating cells

**DOI:** 10.1038/s41598-020-61242-5

**Published:** 2020-03-12

**Authors:** Anne Pora, Sungjun Yoon, Georg Dreissen, Bernd Hoffmann, Rudolf Merkel, Reinhard Windoffer, Rudolf E. Leube

**Affiliations:** 1 Institute of Molecular and Cellular Anatomy, RWTH Aachen University, 52074 Aachen, Germany; 2 Institute of Biological Information Processing 2, Forschungszentrum Jülich, 52425 Jülich, Germany

**Keywords:** Extracellular matrix, Intermediate filaments

## Abstract

Keratin intermediate filaments provide mechanical resilience for epithelia. They are nevertheless highly dynamic and turn over continuously, even in sessile keratinocytes. The aim of this study was to characterize and understand how the dynamic behavior of the keratin cytoskeleton is integrated in migrating cells. By imaging human primary keratinocytes producing fluorescent reporters and by using standardized image analysis we detect inward-directed keratin flow with highest rates in the cell periphery. The keratin flow correlates with speed and trajectory of migration. Changes in fibronectin-coating density and substrate stiffness induces concordant changes in migration speed and keratin flow. When keratinocytes are pseudo-confined on stripes, migration speed and keratin flow are reduced affecting the latter disproportionately. The regulation of keratin flow is linked to the regulation of actin flow. Local speed and direction of keratin and actin flow are very similar in migrating keratinocytes with keratin flow lagging behind actin flow. Conversely, reduced actin flow in areas of high keratin density indicates an inhibitory function of keratins on actin dynamics. Together, we propose that keratins enhance persistence of migration by directing actin dynamics and that the interplay of keratin and actin dynamics is modulated by matrix adhesions.

## Introduction

Cell migration is a highly complex process with relevance for physiological and pathological situations such as embryogenesis, wound healing and metastasis^1^. It is induced by chemical and biophysical cues. The cytoskeleton is at the core of generating effective locomotion by enabling successive steps of protrusion at the cell front and by facilitating the contractile events at the cell rear in parallel with the regulation of cell-matrix adhesions^2^. The cytoskeleton is composed of three major components, namely actin filaments, microtubules and intermediate filaments. The role of actin filaments and microtubules in cell migration has been extensively studied. In contrast, the contribution of intermediate filaments and especially of epithelial keratin intermediate filaments is still poorly understood^3^.

Keratin filaments are the main class of cytoplasmic intermediate filaments in epithelial cells. They belong to a large multigene family encoded by more than 50 genes. Every keratin filament contains equal amounts of type I (acidic) and type II (basic) monomers. Obligatory heterodimers assemble in an antiparallel manner into non-polar tetramers, which further associate laterally and longitudinally to eventually generate 8–12 nm filaments^4–6^.

The effect of keratin expression on migration depends on the keratin isoform, the cell type and the environment^3,7,8^. For example, the knockdown of K8/K18 reduces the invasion capacity of squamous cancer cells^9^ but increases collective cell migration of cancer cells^10^. Depletion of the entire type II keratin gene cluster in mouse keratinocytes prevents keratin filament assembly and induces an increase in migration speed and invasiveness. At the same time, persistence of migration is reduced and cells become more sensitive to mechanical constraints, both of which likely compromises efficient migration in a heterogeneous 3D *in vivo* environment^11–13^.

The structural scaffolding functions of the keratin filament network is contrasted by its highly dynamic properties. A spatially well-defined cycle of assembly and disassembly fuels inward-directed filament motility even in sessile cultured cells. Thus, filaments are nucleated in the cell periphery. These growing filaments move toward the cell center and integrate into the keratin network. Filaments within the network bundle while moving further towards the nucleus where they either become part of a cage-like structure surrounding the nucleus or disassemble into diffusible subunits that are used for another cycle of assembly in the cell periphery^14,15^. It has been suggested that keratin cycling supports rapid cell shape changes to adapt to changing environmental requirements and challenges^15,16^. Nevertheless, the dynamics of keratin filaments have not been investigated in migrating cells so far. Similarly, it is not known how mechanical characteristics of the environment, which are known to modulate cell migration^17^, affect keratin dynamics.

Here, we use primary human keratinocytes to investigate how the distribution and the kinetics of the keratin turnover cycle are affected by cell migration and how this is dependent on the cell’s mechanophysical environment by studying keratinocyte locomotion occurring spontaneously and on defined surfaces with different chemical and physical properties.

## Results

### K5-YFP is a reliable reporter to measure keratin dynamics in migrating normal human epidermal keratinocytes

It has been suggested that the keratin cycle of assembly and disassembly supports rapid shape changes of epithelial cells^15^. However, to date the keratin turnover cycle has not been examined during cell migration. To do this, spontaneously migrating normal human epidermal keratinocytes (nHEKs) from neonatal foreskin were used. They were seeded at very low density (~5 000 cells/cm^2^) and were analyzed after two days. We would like to stress that this paradigm differs from the sheet-like migration of epidermal monolayers that is typically encountered *in vivo*, e.g. during wound healing^18^. Experiments were performed at passages 2–4 to avoid cell differentiation^19^. When seeded on fibronectin-coated glass, single nHEKs migrated spontaneously adopting a characteristic D-shape with a large lamellipodium and multiple filopodia at the cell front (red arrows; Supplementary Fig. S1A) and long retraction fibers at the back (blue arrows; Supplementary Fig. S1A). Gel electrophoretic separation of high salt cytoskeletal extracts prepared from nHEKs revealed multiple major polypeptides (Fig. S1B). Immunoblotting identified the major polypeptide bands as the expected basal keratinocyte-specific K5 and K14, the hyperproliferation-associated K6, K16 and only a little K17, and, in addition, small amounts of the differentiation-dependent K1 and K10 all of which are typically found in foreskin keratinocytes (Supplementary Fig. S1C)^20^. Immunohistology further revealed that K5, K6, K14, K16 and K17 are produced in all cells, whereas only trace amounts of K1 and K10 could be detected in a few cells (Supplementary Fig. S1D–J).

K5-YFP-encoding constructs were prepared to monitor keratin dynamics. Fluorescence microscopy of transiently transfected nHEKs (referred to as K5-YFP nHEKs) revealed a typical cytoplasmic keratin network and co-localization of the fluorescent transgene product with the endogenous keratin network throughout the cells (Supplementary Fig. S2A-A”). Side-by-side comparison of transfected and non-transfected cells further suggested that the levels of keratins were not drastically elevated in the transfected cells (Fig. S2B,B’). Furthermore, expression of K5-YFP did not visibly affect the morphology of migrating nHEKS or their unique and highly conspicuous keratin network organization (Supplementary Fig. S2B–D). The keratin network was concentrated around the nucleus (white arrows in Supplementary Fig. S2C,D) with bilateral extensions of keratin bundles along the retracting part of the cell (red arrows in Supplementary Fig. S2C,D). Filament density and bundle thickness decreased towards the cell front.

Given the reported effects of keratins on cell migration^7,8^, we assessed the consequences of K5 overexpression on nHEK migration. In comparison to nHEKs transfected with a construct coding for cytoplasmic YFP, K5-YFP nHEKs migrated slower (0.66 ± 0.0026 µm.min^−1^ versus 0.89 ± 0.0035 µm.min^−1^; Supplementary Fig. S2E). On the other hand, increased persistence was noted in K5-YFP nHEKs as evidenced by an elevated directionality ratio (Supplementary Fig. S2F).

### The keratin flow pattern in migrating keratinocytes differs between cell front, center and back with respect to speed and direction of keratin flow

To examine the keratin flow in migrating nHEKs, confocal time-lapse fluorescence microscopy (30 min recordings, 1 image per min) was performed on single K5-YFP nHEKs grown on fibronectin-coated glass (Movie 1). Fluorescence was recorded in the focal plane of the ventral part of the cell, which contains most of the keratin network (Supplementary Fig. S2D; see also^14^). This focal plane is slightly on top of the cortical actin network.

**Figure 1:**
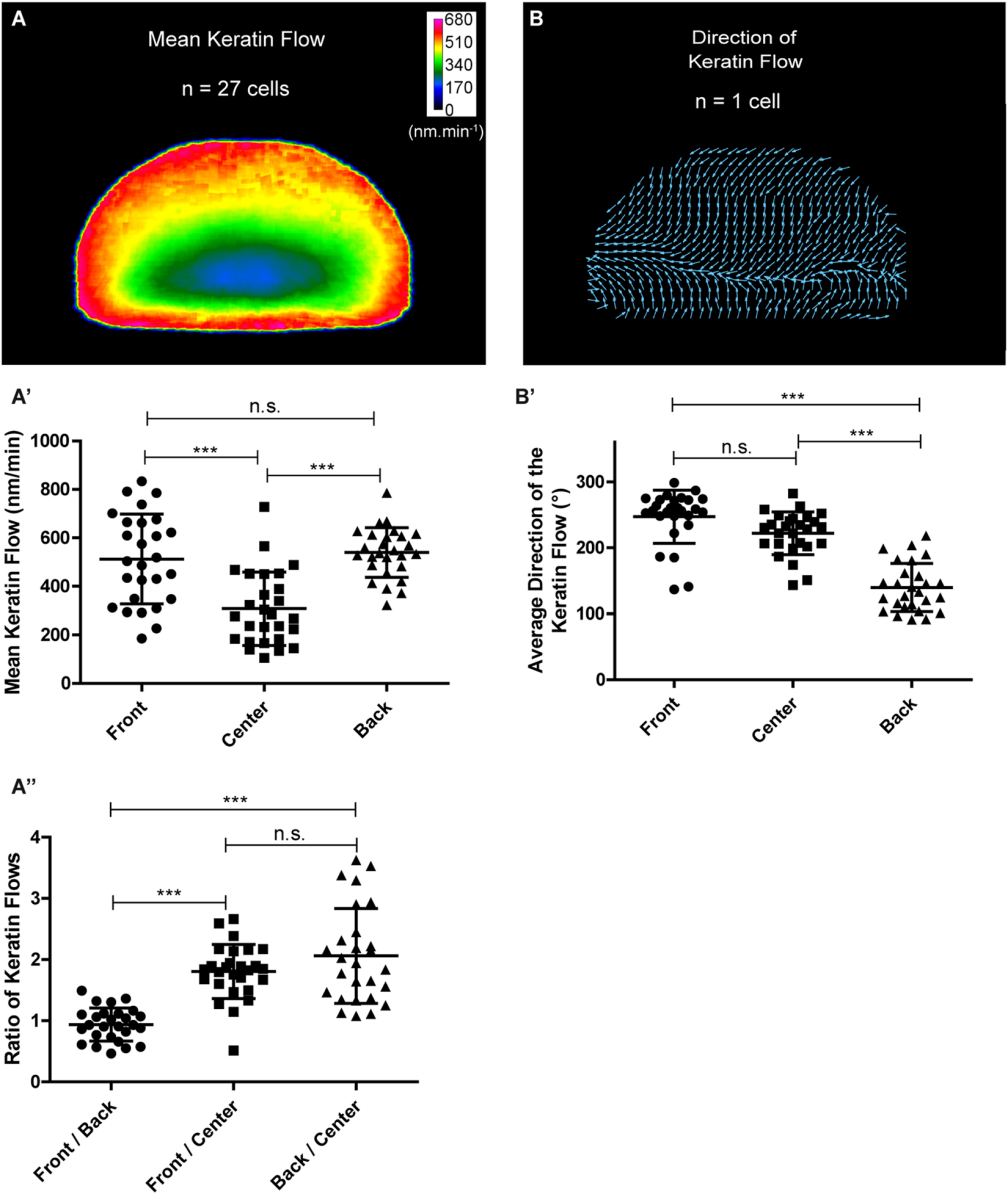
The keratin flow in migrating nHEKs has a well-defined spatial distribution. Data were extracted from live-cell confocal fluorescence images (objective 63 x) of 27 transiently transfected nHEK K5-YFP cells migrating on fibronectin-coated glass (30 min recording, 1 image.min^−1^; see also Supplementary Fig. S4). (**A-A”**) The mean speed of the keratin flow is depicted as a heat map (**A**) and as column scatter plots of the cell front, center and back (**A’**). The ratios of the mean speeds of keratin flow determined in different parts of the cells are shown in (**A”**). The heatmaps were obtained after shape normalization. Highest flow is found at the periphery of the cell, while lowest flow was found close to the nucleus. For (**A’**,**A”**), ANOVA was used for statistical analysis (P < 0.0001) followed by Tukey’s test between all pairs of columns. (**B-B’**) Vector map of a single cell recording and column scatter plots representing the direction of keratin flow. 90° is defined as the direction of migration. Kruskal-Wallis test was used for statistical analysis (P < 0.0001) followed by Dunn’s multiple comparison test between all pairs of columns. The flow is retrograde in the front and center, and in the direction of migration at the back end. n.s., not significant. The figure is modified from^57^.

Quantitative image analysis was carried out using the cross-correlation based program CMove (for details see Materials and Methods). This program determined keratin flow defined as the speed of keratin patterns in the reference frame of the cell (Supplementary Fig. S3). To compare keratin flow patterns in different migrating cells, the results were normalized to a standardized D-like shape. Images of the fluorescence patterns prior to normalization are shown for the first set of cells in Supplementary Fig. S4. The figure also depicts the fluorescence-derived shape used for normalization and the center of mass. The resulting heat- and vectormaps revealed specific flow patterns with high and inward directed flux from the peripheral cytoplasm to the nucleus (Fig. 1A,B). The maximum speed was up to 0.9 µm.min^−1^, which is 3–4 times higher than the maximum values determined previously in other sessile cells even after EGF stimulation^14^.

To further dissect the keratin flow pattern, the cell area was arbitrarily split into front, center and back (Supplementary Fig. S5). Highest flow was found in the cell periphery, while lowest values were found in the cell center close to the nucleus (Fig. 1A,A’). Figure 1A” further demonstrates that the flow in the periphery was equally elevated in the front and back by a factor of ≈2 in comparison to the cell center. Analysis of the direction of the keratin flow showed that it is retrograde in the front of the cell and anterograde in the back of the cell (Fig. 1B,B’).

### High keratin flow correlates with high migration speed

**Figure 2:**
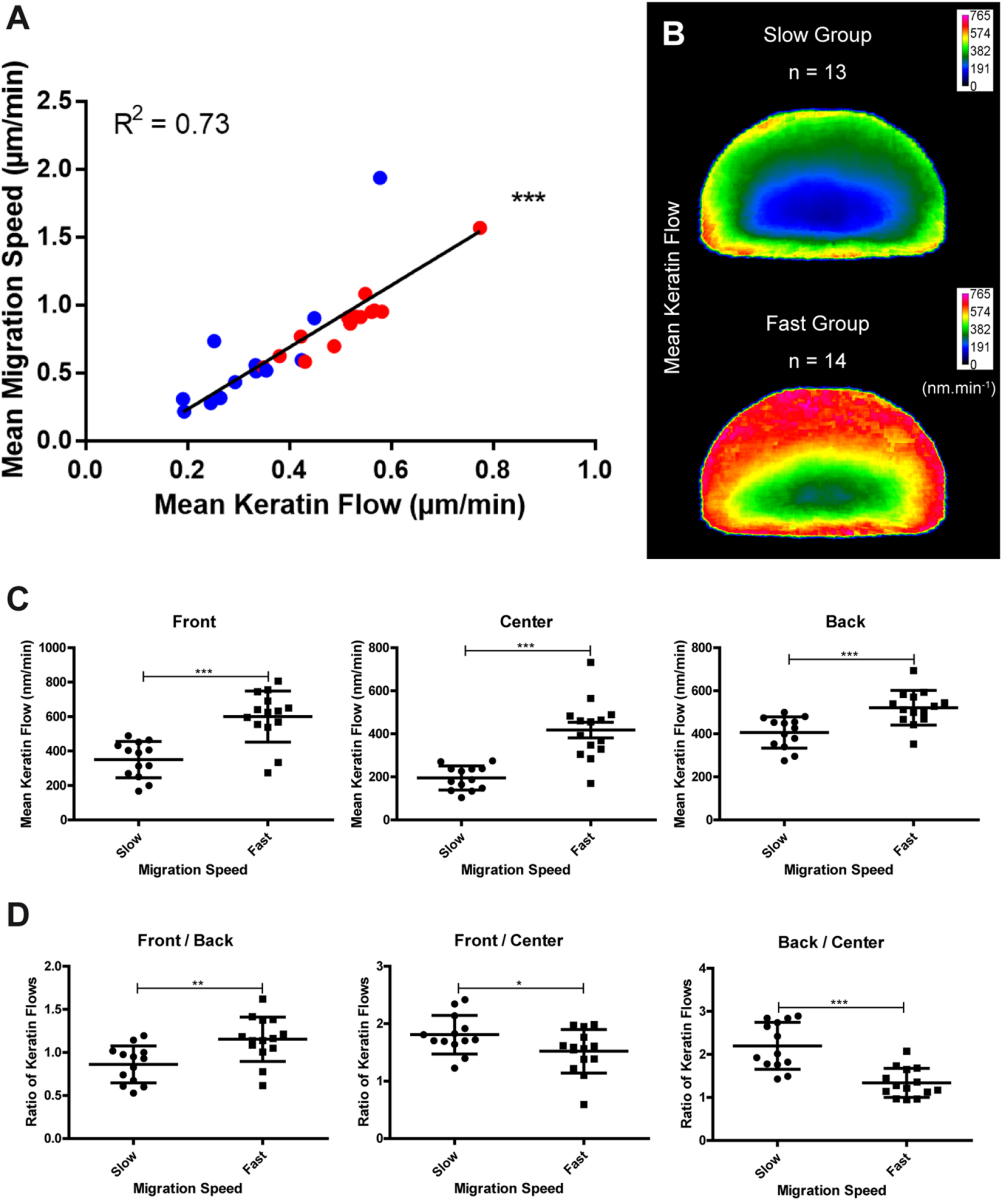
Higher migration speed correlates with increased keratin flow. Data were extracted from live-cell confocal fluorescence images (same as those used for Fig. 1) of nHEKs transiently transfected with K5-YFP. (**A**) Graph of the mean migration speed in relation to the mean keratin flow. Each dot represents one cell (n = 27). Higher keratin flow correlates with higher migration speed and the increase appears linear. Statistical analysis was performed using Spearman correlation (P < 0.0001, R² = 0.73). Red and blue denotes migrating cells with high and low directionality, respectively. (**B–D**) The cells were grouped as slow (n = 13) comprising cells with a migration speed < 0.65 µm.min^−1^ and fast (n = 14) comprising cells with a migration speed > 0.65 µm.min^−1^. (**B**) Heat maps of the mean normalized keratin flow in the slow (top) and fast (bottom) group after shape normalization. (**C**) Column scatter plots of the mean keratin flow in the cell front, center and the back of both groups. (**D**) Column scatter plots of the ratios between the mean keratin flow in different areas of fast and slow moving nHEKs. Note that the two groups have significantly different mean migration speeds. There is an overall increase in the keratin flow between both groups. The strongest increase is found in the cell center, the lowest in the back of the cell. Statistical analysis was performed using unpaired Student t-test (P < 0.0001 in D (with Welch correction in center area); P = 0.0037 in D (front/back); P = 0.0457 in D (front/center); P < 0.0001 in D (back/center)). The figure is modified from^57^.

For each cell, the mean keratin flow and the mean migration speed were calculated. This showed that higher keratin flow correlated with higher migration speed (Fig. 2A). Within the limits of our measurements, the relation between migration speed and keratin flow appeared linear. The extrapolated keratin flow speed for a sessile cell according to the least squares fit analysis is ≈0.12 µm.min^−1^, which is consistent with the values determined previously for sessile A431- and HaCaT-derived cells^14^.

To further dissect the effect of migration speed on keratin flow at the subcellular level, cells were sorted into a slow group (n = 13) and a fast group (n = 14). For each group, the normalized mean keratin flow was calculated for heatmap presentation (Fig. 2B). The values of the keratin flow in the front, center and back of the cells were then compared between the slow and fast group. This showed that the keratin flow was higher in faster moving cells presenting significantly higher keratin flow in all three zones as compared to the corresponding zones in the slow group (Fig. 2C). The highest increase was noted in the cell center (Fig. 2D).

### The keratin flow speed mirrors the trajectory of cell migration

**Figure 3:**
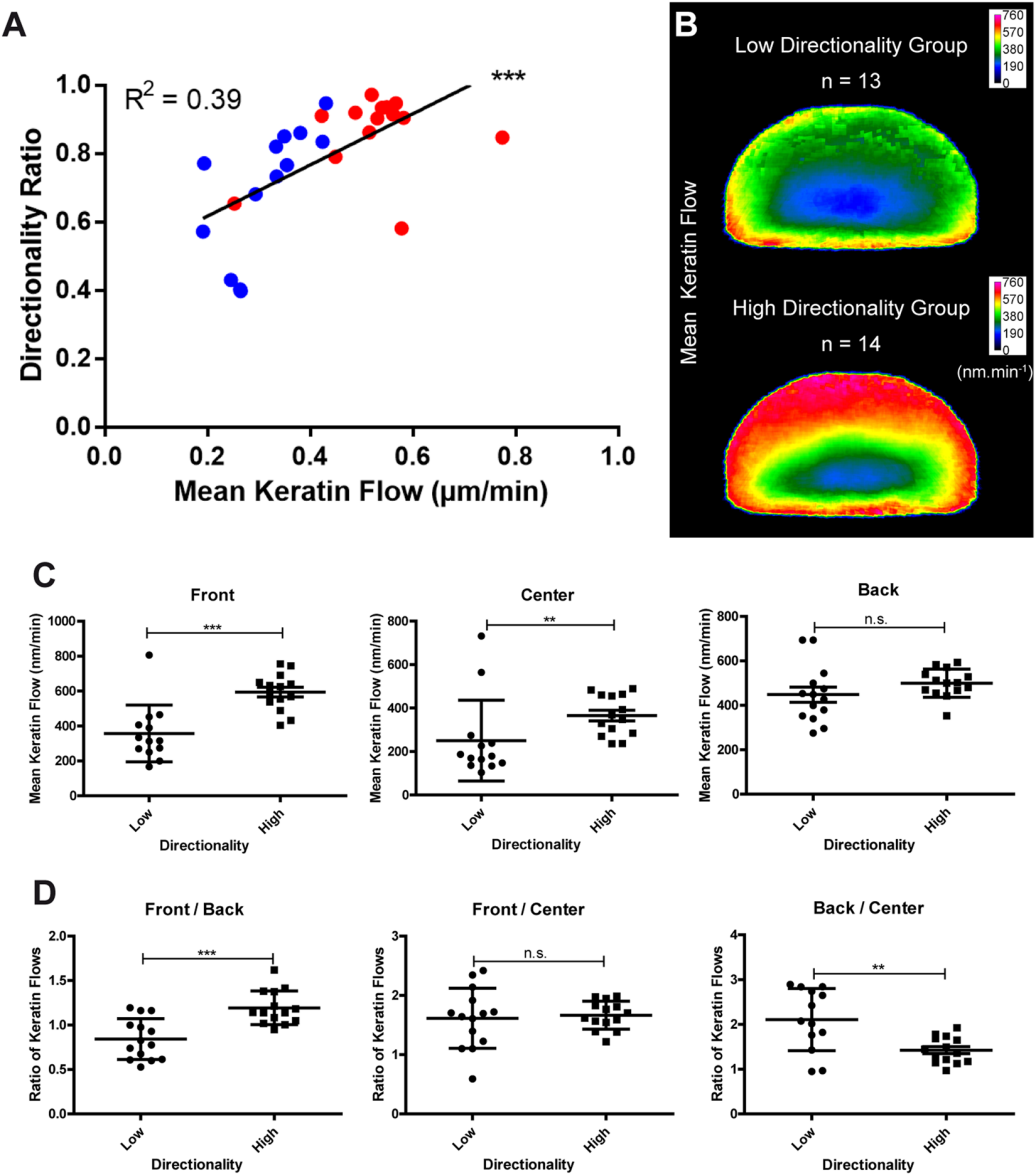
Higher directionality ratio of migration correlates with increased keratin flow. Data were extracted from live-cell confocal images (same as those used for Figs. 1 and 2) of transiently transfected K5-YFP nHEKs. (**A**) Graph of the directionality ratio in relation to the mean keratin flow. Each dot represents one cell (n = 27). Higher keratin flow correlates with higher directionality ratio. Statistical analysis was performed using Spearman correlation test (P = 0.0001, R² = 0.3896). Red and blue denote fast and slow migrating cells, respectively. (**B–D**) The cells were divided into a low directionality group (n = 13) comprising cells with a directionality ratio < 0.84 and a high directionality group (n = 14) comprising cells with a directionality ratio > 0.84. (**B**) Heat maps of the mean keratin flow in the low (top) and high directionality groups (bottom) after shape normalization. (**C**) Column scatter plots of the mean keratin flow in the cell front, center and the back of both groups. The two groups show significantly different directionality ratios. There is an overall increase in keratin flow in the high directionality versus the low directionality group. The strongest increase is found in the front and center of the cell. The following statistical tests were used: Mann-Whitney test (P = 0.0005 for front area in D; P = 0.0053 for center area in C), unpaired Student t-test (P = 0.532 for back area in C; P < 0.0001 for front/back in D), Student t-test with Welch’s correction (P = 0.9424 for front/center in D; P = 0.0051 for back/center in C). The figure is modified from^57^.

To examine the relationship between keratin flow and the trajectory of cell migration, the directionality ratios were calculated for each cell and compared to the keratin flow speed. The directionality ratio is a measure of migration straightness and hence persistence of cell migration. Faster keratin flow correlated with higher directionality ratios (Fig. 3A). The fast and slow cells are color-coded as red and blue dots, respectively, in Fig. 3A showing a correlation between overall migration speed, directionality and mean keratin flow. Similarly, color coding cells with high and low directionality in Fig. 2A shows the same correlation. To examine the effect of an increase in persistence of cell migration on the keratin flow at the subcellular level, cells were sorted into a low directionality group (n = 13) and a high directionality group (n = 14).

The averaged keratin flow on normalized geometry was then calculated and used for the heatmaps in Fig. 3B and the column scatter plots in Fig. 3C,D. A significantly higher keratin flow was found in the front and the center area for the group with higher directionality. The ratio between the flows in the front and the back of the cells was increased for the high directionality group whereas the ratio between the front and center was unchanged and the ratio between the back and center was decreased in comparison to the group with lower directionality showing that the increase in keratin flow associated with higher directionality ratio was strongest in the center and front.

**Figure 4:**
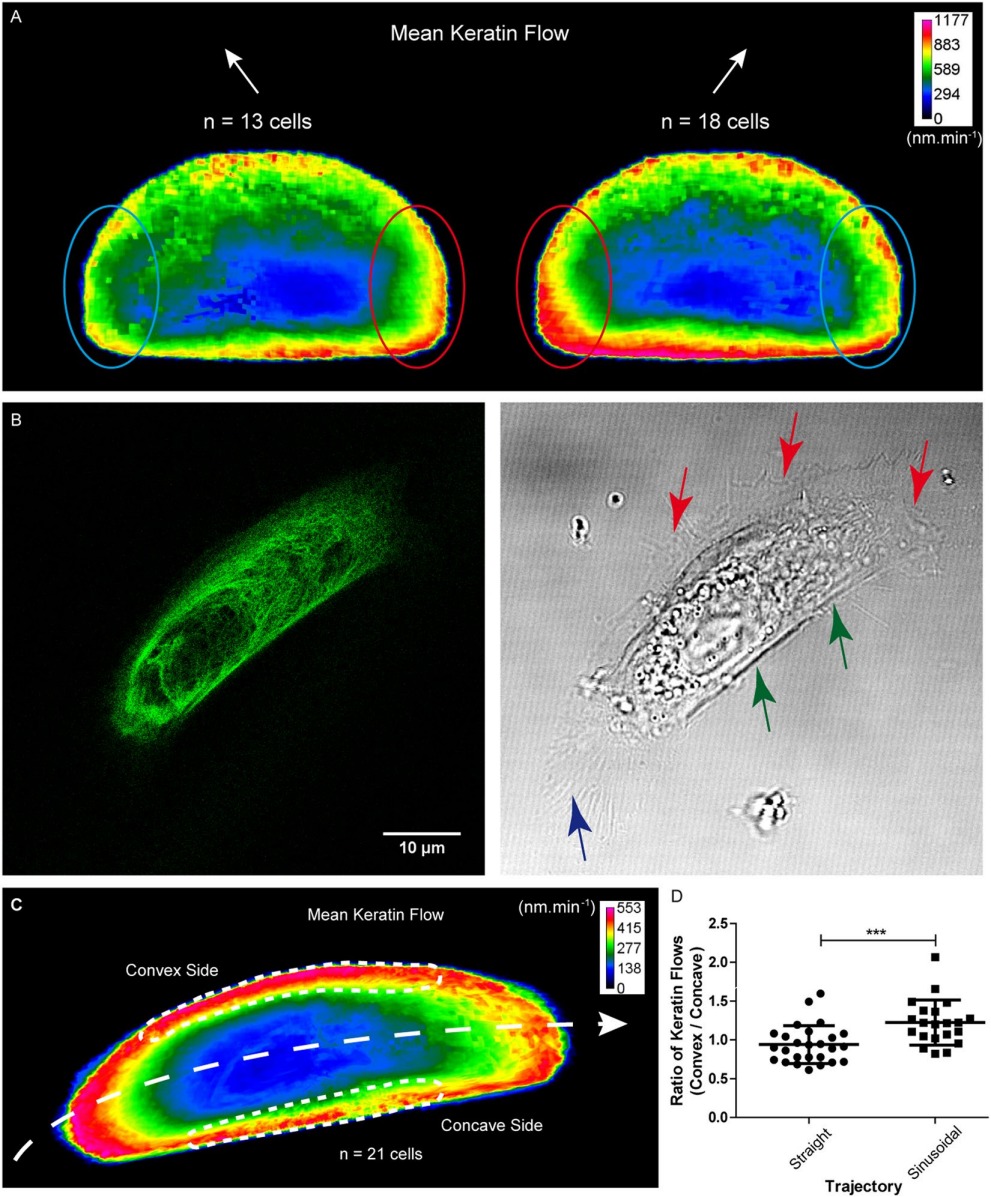
Change in direction of migration induces symmetry break in keratin flow patterns. Keratin flow was determined in K5-YFP nHEKs in different situations. **(A)** Fluorescence recordings of nHEKs producing K5-YFP and migrating on fibronectin-coated glass slides were selected for cells that either turned left or right during 30 min confocal time-lapse imaging (n = 11 with 60 s intervals and n = 20 with 120 s intervals) as judged from CMove analysis. The heat maps depict the shape-normalized mean keratin flow patterns of 13 cells turning left and 18 cells turning right. Note that areas with higher flow are located directly opposite to the new direction of migration, i.e. at the right back corner of cells turning left and vice versa (compare corresponding areas at the left and right back of the cells circled in red (higher flow) and blue (lower flow)). (**B**) The live-cell confocal fluorescence and corresponding phase contrast image (objective 63 x) of an K5-YFP nHEK migrating toward the right on a micropatterned fibronectin-coated sinusoidal stripe (width 15 µm, curvature 0.02 µm^−1^) is taken from corresponding Movie 2. The elongated cell adapts to the line width and the nucleus is shifted towards the back. Highly dynamic filopodia and lamellopodia are seen at the front and convex cell margins (red arrows) whereas the concave margin is straight (green arrows) and the cell rear extends long retraction fibers (blue arrow). (**C**) Heat map of the mean normalized keratin flow derived from fluorescence recordings of 21 K5-YFP nHEKs migrating on a sinusoidal stripe after shape normalization. Highest flow is found in the front and convex margin. (**D**) Column scatter plots depicting the ratio between the average keratin flow in the convex and concave part of cells migrating on sinusoidal stripes (n = 21). For comparison K5-YFP nHEKs migrating on straight stripes (width 15 µm; n = 26) were imaged. Statistical analysis was performed using Mann-Whitney test (P = 0.0007). Higher asymmetry in the keratin flow is seen for cells on sinusoidal than on straight stripes. The figure is modified from^57^.

Next, migrating K5-YFP nHEKs were sorted into left turning cells (n = 13) and right turning cells (n = 18). In each case, a symmetry break was observed for the keratin flow pattern in the lateral back part of the cells with elevated keratin flow on the side opposite to the direction of migration (compare areas circled in red and in blue in Fig. 4A).

To standardize experimental conditions, K5-YFP nHEKs were seeded on micropatterned coverslips containing fibronectin-coated sinusoidal stripes (15 µm width, curvature 0.02 µm^−1^). nHEKs adopted an elongated shape (Fig. 4B; Supplementary Fig. S6A). They migrated spontaneously with speeds ranging between 0.2 µm.min^−1^ and 1 µm.min^−1^. Keratin dynamics were recorded by confocal time-lapse fluorescence microscopy (Movie 2) and analyzed with the help of CMove. The results were shape normalized to a flattened D-shape and the average flow was calculated. The heatmap in Fig. 4C shows that higher keratin flow occurred on the convex side in comparison to the concave side. For comparison, cells were also grown on straight 15 µm-wide fibronectin-coated stripes (Supplementary Fig. S6B) and analyzed similarly (Movie 3). Quantitative assessment revealed symmetric keratin flow in cells moving on straight stripes in contrast to the asymmetric keratin flow pattern of cells moving on sinusoidal stripes (Fig. 4D). Together, the analyses of nHEKs migrating on defined micropatterns fully confirmed the results obtained for freely migrating nHEKs. We therefore conclude that keratin dynamics are finely co-regulated with the speed and trajectory of cell migration.

### Confinement of migrating normal human epidermal keratinocytes reduces keratin flow disproportionally

**Figure 5:**
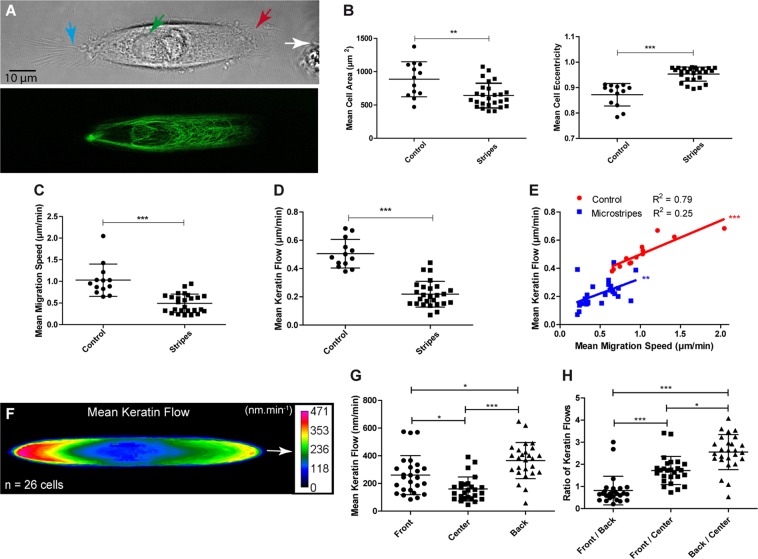
Confinement impedes migration speed and keratin flow. K5-YFP nHEK migration was restricted to micropatterned 15 µm wide fibronectin-coated stripes. As control, K5-YFP-expressing nHEKs were seeded on fibronectin-coated coverslips that were prepared by deep UV illumination exactly like the micropatterned coverslips but covering the entire glass surface of the coverslip (i.e. without mask). Fluorescence images were recorded by confocal laser microscopy (objective 63 ×, 1 image.min^−1^ for 30 min; n = 26 for cells on micropattern; n = 13 for control cells). (**A**) Fluorescence and corresponding brightfield image of a K5-YFP nHEK migrating on a fibronectin-coated stripe (see corresponding Movie 3). The cell is elongated and the nucleus (green arrow) is shifted towards the back. The front of the cell (red arrow) is rich in lamellipodia and filopodia, while the back (blue arrow) contains multiple retraction fibers. (**B**) Column scatter blots depict the effect of confinement for mean cell area and mean cell eccentricity. Unpaired Student t-test (P = 0.018 for mean cell area; P < 0.0001 for mean cell eccentricity). (**C**) Graphical representation of the stripe-induced pseudo-confinement on mean migration speed. Mann-Whitney test (P < 0.0001). (**D**) Graphical representation of the mean keratin flow depending on stripe-induced confinement. Unpaired Student t-test (P < 0.0001). (**E**) Graph depicting the relationship between the mean migration speed and mean keratin flow with or without confinement. For a given migration speed, keratin flow is slower for cells forced to migrate on stripes than for free migrating cells. Pearson correlation (Control: P < 0.0001, R² = 0.7859; Stripes: P = 0.009, R² = 0.2509). (**F**) Heatmap representing the mean speed of the keratin flow after shape normalization in K5-YFP nHEKs migrating on a stripe. (**G**) Column scatter plots of the speed of the keratin flow in the front, center and back of cells migrating on stripes. (**H**) Column scatter plots of the ratios between the speed of the keratin flow in different areas of cells migrating on stripes. For (**G,H**) Kruskal-Wallis test was used for statistical analysis (P < 0.0001) then Dunn’s test between all pairs of columns. The figure is modified from^57^.

Confinement has been shown to affect migration^21,22^. We therefore wanted to find out, how this might modulate keratin flow patterns. To do this, we studied K5-YFP nHEKs on straight 15 µm-wide fibronectin-coated stripes (see above). Similar to cells moving on the sinusoidal stripes, the cells drastically elongated with a leading edge characterized by lamellipodia and filopodia and a cell rear with typical retraction fibers (compare Fig. 4B with 5A; see also Supplementary Fig. S6, Movie 3). As a control, cells were grown under identical conditions on coverslips that had been treated in the same way as micropatterned coverslips, but without a mask. The cell eccentricity was strongly increased in nHEKs moving on the stripes in comparison to those freely-moving on the evenly coated surface. But the cell area was reduced (Fig. 5B). The migration speed was also reduced on stripes when compared to the control (Fig. 5C). In accordance, keratin flow was slower on stripes in comparison to the control (Fig. 5D). Remarkably, we observed that for a given migration speed, the corresponding keratin flow was consistently slower for cells migrating on stripes than for freely-moving cells (Fig. 5E). The heatmap in Fig. 5F shows that the keratin flow was highest in the back of the cells moving on straight stripes. The somewhat slower flow at the cell front was still significantly higher than in the cell center (Fig. 5F–H).

### Extracellular matrix coating density affects keratin flow

**Figure 6:**
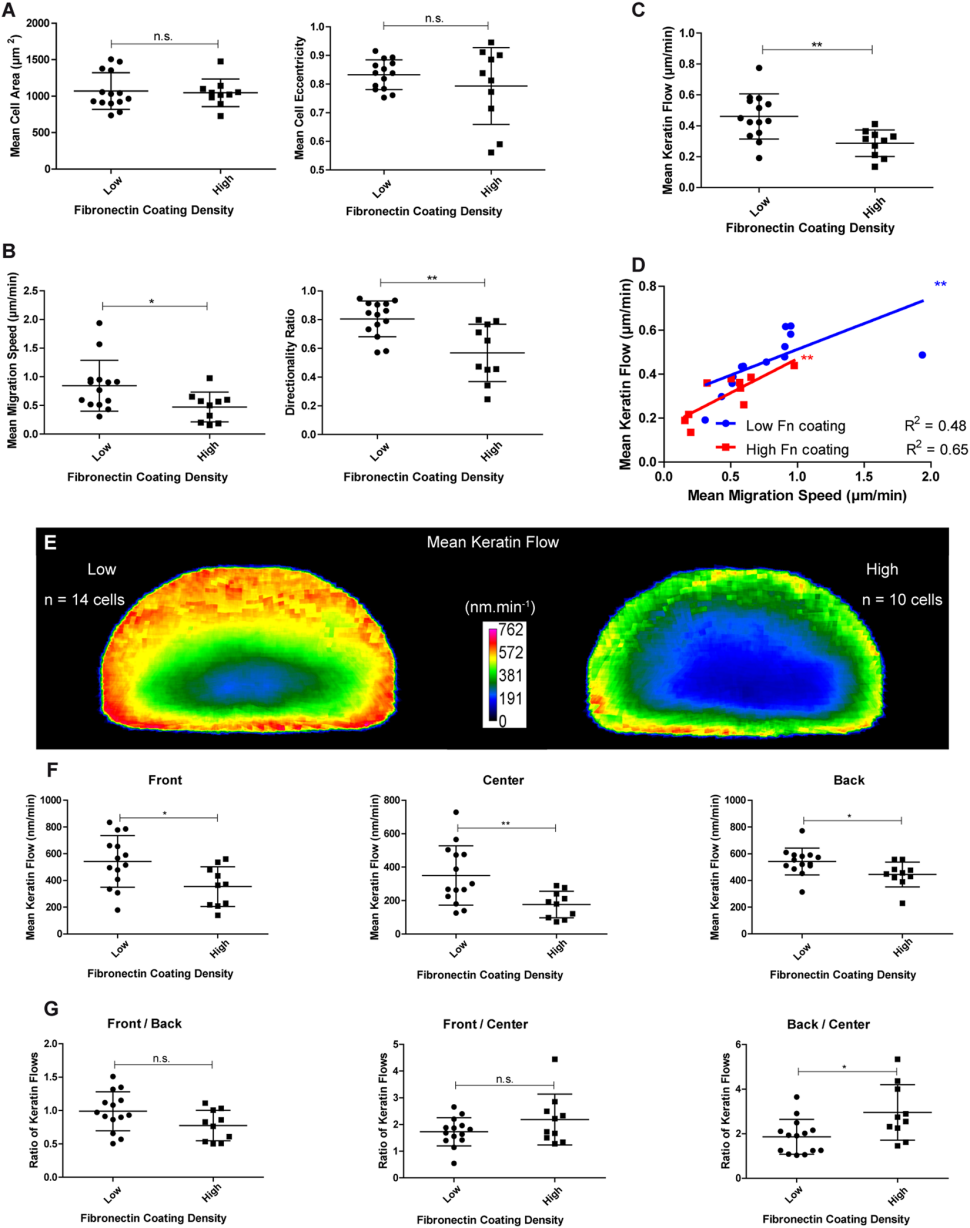
Increased ECM coating density induces a decrease in migration speed and keratin flow. Data were extracted from confocal images (30 min recordings, 1 image.min^−1^; objective 63 x) of transiently transfected K5-YFP nHEKs migrating on glass with low (n = 14) or high fibronectin coating density (n = 10). (**A**) Column scatter plots show fibronectin density in relation to mean cell area (left) and mean cell eccentricity (right). Unpaired Student t-test for mean cell area (P = 0.8011) and Student t-test with Welch’s correction for mean cell eccentricity (P = 0.3966; n.s., not significant). Note that the cell morphology is not affected by different coating densities. (**B**) Graphical representation of migration characteristics in relation to coating density. At left, mean migration speed is shown (Mann-Whitney test; P = 0.0434); at right, the directionality ratio is depicted (unpaired Student t-test; P = 0.0017). An increase in coating density correlates with a decrease in migration speed and directionality. (**C**) Depicts relationship between keratin flow and fibronectin coating density. Unpaired Student t-test (P = 0.0029). (**D**) Graph of mean migration speed versus mean keratin flow. For a given migration speed, the corresponding keratin flow is similar irrespective of the coating density. Pearson correlation (low fibronectin coating density: P = 0.0063, R² = 0.4765; high fibronectin coating density: P = 0.0047, R² = 0.6530). (**E**) Heat maps showing the mean keratin flow in shape-normalized nHEKs migrating on low and high density fibronectin (n = 14 and n = 10, respectively). (**F**) Quantification of the effect of fibronectin coating densities on keratin flow in the cell front, center and back. Reduction is seen in all cell regions with the strongest decrease in the cell center. Unpaired Student t-test (P = 0.0174; front), Student t-test with Welch’s correction (P = 0.0044; center) and Mann-Whitney test (P = 0.0109; back). (**G**) The column scatter plots show keratin flow ratios in different cell regions at low and high fibronectin coating density. Unpaired Student t-test (P = 0.0684; front/back), Mann-Whitney test (P = 0.3641; front/center), and unpaired Student t-test (P = 0.0148; back/center). The figure is modified from^57^.

To further examine the relationship between cell migration and keratin flow patterns, we studied the impact of extracellular matrix coating density, which is known to affect speed of cell migration^1,23^. To this end, K5-YFP nHEKs were seeded on glass coverslips coated with fibronectin at low and high density (details in Material and Methods). Keratin dynamics were then measured in migrating cells as described above. The mean cell area and cell shape were unchanged in both conditions (Fig. 6A). But the migration speed and the directionality ratio were lower in cells on high fibronectin density compared to cells on low fibronectin density (Fig. 6B). The keratin flow was also lower when the coating density was high (Fig. 6C). We further observed that the keratin flow for a given migration speed was the same independent of the fibronectin concentration (Fig. 6D). The heatmaps in Fig. 6E revealed that the keratin flow was reduced in the entire cells on substrates with higher coating density. Quantification showed that keratin flow was decreased in the cell front, center and back (Fig. 6F). The ratios between the flows in the front and the back and between the front and the center of the cells were unchanged whereas the ratios between the back and the center were increased on high fibronectin coating density (Fig. 6G) showing that the decrease in keratin flow associated with higher coating density is strongest in the cell center.

### Decreased substrate stiffness increases keratin flow

**Figure 7:**
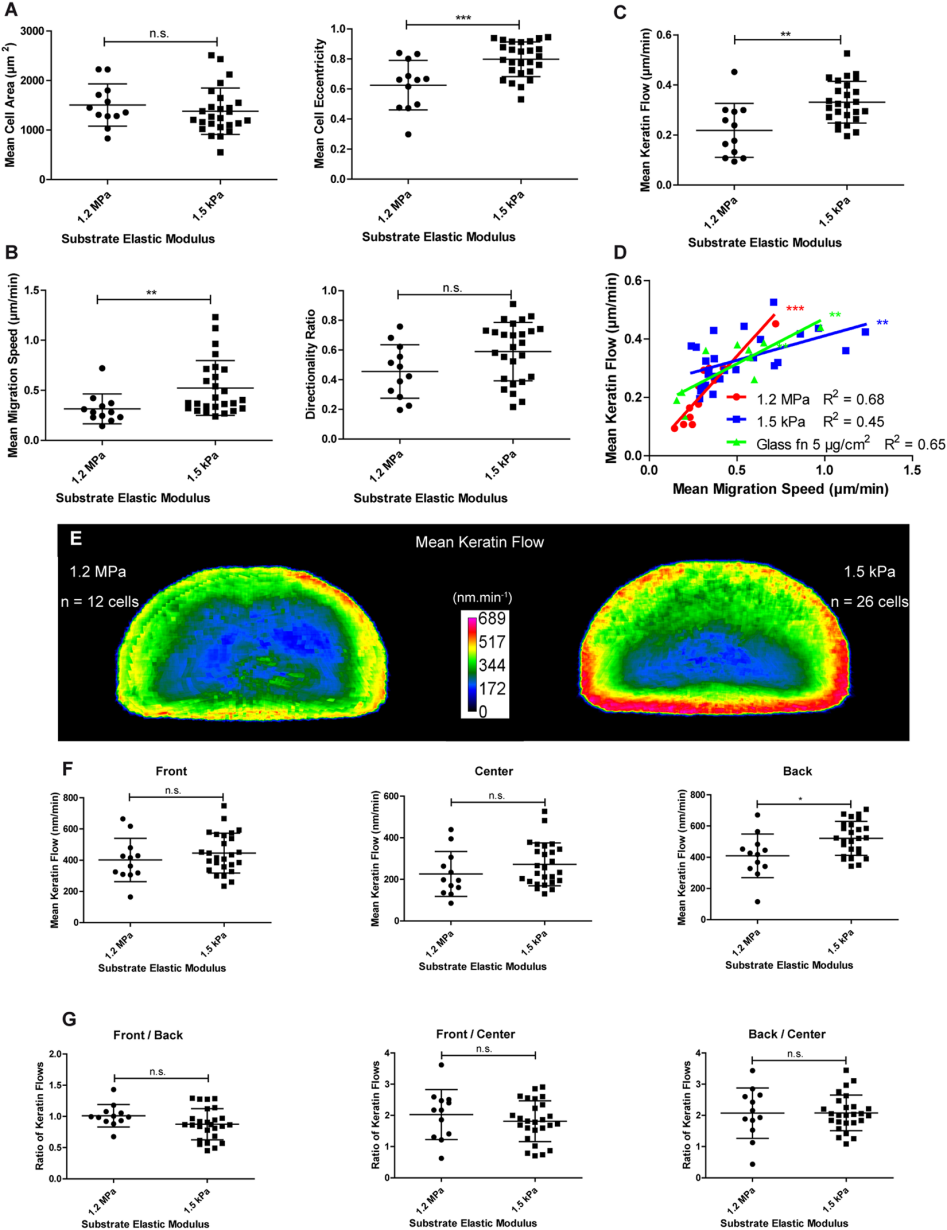
Decreased substrate stiffness induces an increase in migration speed and keratin flow. Data were extracted from confocal images (30 min recordings, 1 image.min^−1^; objective 63 x) of transiently transfected K5-YFP nHEKs migrating on elastomeric substrates with high (1.2 MPa; n = 12) and low stiffness (1.5 kPa; n = 26). (**A**) Column scatter plots show the relationship between substrate stiffness and mean cell area (left) and mean cell eccentricity (right). Unpaired Student t-test (P = 0.4327 for mean cell area; P = 0.0007 for mean cell eccentricity). (**B**) Graphical representation of migration characteristics depending on the elastic modulus of the substrate. Mean migration speed is shown at left (Mann-Whitney test; P = 0.0035), directionality ratio at right (unpaired Student t-test; P = 0.0524). (**C**) Column scatter plots of the mean keratin flow depending on the elastic modulus of the substrate. Unpaired Student t-test; P = 0.0012). (**D**) Graph shows the relationship between mean migration speed and mean keratin flow in cells grown on substrates with different elastic moduli. Pearson correlation (glass coated with high fibronectin (fn): P = 0.0047, R² = 0.6530 [n = 10]; 1.2 MPa: P < 0.0001, R² = 0.8434; 1.5 kPa P = 0.0035, R² = 0.3034). (**E**) Heat maps of the mean keratin flow in shape-normalized nHEKs migrating on PDMS substrates with an elastic modulus of 1.2 MPa (n = 12) or 1.5 kPa (n = 26). (**F**) The column scatter plots show the effect of the elastic modulus of the substrate for keratin flow in the cell front, center and back. Unpaired Student t-test (P = 0.3434, front; P = 0.2165, center; P = 0.0105, back). (**G**) The column scatter plots show the ratios of keratin flow in different cell regions on substrates with high (1.2 Mpa) and low stiffness (1.5 kPa). Unpaired Student t-test (P = 0.1014, front/back; P = 0.3876, front/center, P = 0.9756, back/center). On softer substrates the increase in keratin flow is clearly detectable in the back of the cells. The figure is modified from^57^.

It is known that substrate stiffness affects cell migration^1,24–28^. To study its impact on keratin flow, K5-YFP nHEKs were seeded on fibronectin-coated (high density) elastic substrates with high stiffness (1.2 MPa) and low stiffness (1.5 kPa). The cells spread equally on both substrates (Fig. 7A) but the cell shape was modified. A higher eccentricity was found for cells on soft substrates because of increased elongation of cells perpendicular to the direction of migration (Fig. 7A). The migration speed and the directionality ratio of cells migrating on soft substrates were higher than for cells migrating on stiff substrates (Fig. 7B). Keratin flow was higher on soft than on stiff substrates (Fig. 7C). We further noted that for a given migration speed the associated keratin flow was the same irrespective of substrate stiffness (Fig. 7D). Subcellular mapping further showed an overall increase of the keratin flow with the highest increase in the cell back (Fig. 7E,F). The ratios between the keratin flows in the front, center and back of the cells were the same (Fig. 7G).

### Keratin flow lags behind actin flow in migrating keratinocytes

**Figure 8:**
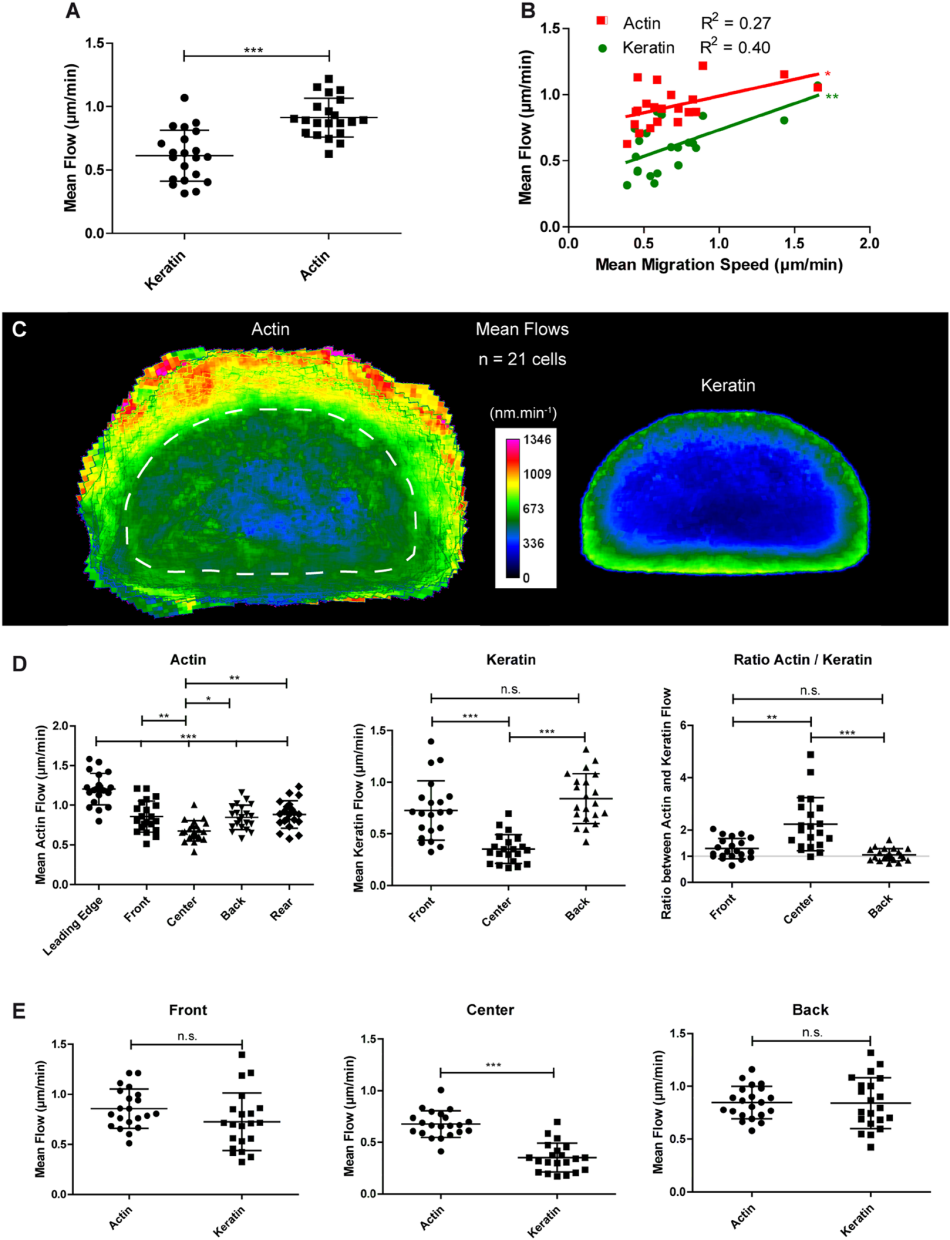
Actin and keratin interact dynamically during migration. Data were extracted from live-cell confocal images (objective 63 x) of nHEKs transiently co-transfected with K5-YFP and LifeAct-DsRed constructs (n = 21) migrating on fibronectin-coated glass (30 min recording, 2 images.min^−1^; see also Movie 4). (**A**) Column scatter plot of the mean keratin and actin flow. Unpaired Student t-test (P < 0.0001). (**B**) Graph showing the mean actin and keratin flow in relation to the migration speed. Pearson correlation (for keratin P = 0.0022, R^2^ = 0.3974 and for actin P = 0.0152 and R^2^ = 0.2724). (**C**) Heat maps of the mean actin and keratin flows in shape-normalized migrating nHEKs. The signal shown for the actin flow outside the normalized shape corresponds to peripheral areas where actin can be found but no keratin (dotted line demarcates area with keratins). In these areas, the average flow was calculated only over the number of cells in which actin was detectable. (**D**) Left: Graphical representation of the speed of actin flow in five different areas of the cell. ANOVA (P < 0.0001) followed by Tukey’s test on all pairs of columns. Middle: Graphical representation of the speed of keratin flow in three different areas of the cell. ANOVA (P < 0.0001) followed by Tukey’s test on all pairs of columns. Right: Graphical representation of the ratio between actin and keratin flows in three different areas of the cells where both cytoskeletal components are detected. Kruskal-Wallis test (P < 0.0001) followed by Dunn’s test. (**E**) Column scatter plots of the actin and the keratin flow in the front, center and back of cells where both cytoskeletal components are detected. Unpaired Student t-test, left (P = 0.0932); unpaired Student t-test, middle (P < 0.0001); t-test with Welch’s correction, right (P = 0.9412). The figure is modified from^57^.

To test the idea, that the mechanical microenvironment exerts its effect on the keratin system via focal adhesions and their associated actin filaments^29,30^, we quantified focal adhesion density by automated image analysis in relation to fibronectin coating density and substrate stiffness (Supplementary Fig. S7A, B). As expected, focal adhesion density was increased on high fibronectin density and increased substrate stiffness. To investigate the relationship between actin and keratin dynamics in migrating nHEKs, cells were then doubly transfected with the K5-YFP-encoding construct and LifeAct-RFP for subsequent time-lapse recordings (30 min recording period, 2 images per min, Movie 4). Actin and keratin dynamics were measured using CMove as specified in Materials and Methods and Supplementary Fig. S8. The mean actin flow was in the expected range (0.913 ± 0.154 μm/min) when compared to values reported in the literature using other methods (see, e.g.^31,32^). When averaging the flows in entire cells, actin flow was faster than keratin flow (Fig. 8A). Moreover, for a given migration speed, the actin flow was higher than the keratin flow (Fig. 8B). To study actin and keratin flows at the subcellular level, each flow pattern was normalized separately (details in Supplementary Figs. S5, S8). The resulting heatmaps revealed that actin flow was particularly high in the most peripheral part of the cell front that is devoid of keratin (Fig. 8C). Actin flow rates dramatically decreased at the border to the area with keratins (dashed line in Fig. 8C). Within the overlap regions actin and keratin flow patterns were highly similar with comparable speed and direction. Yet, keratin flow was always slightly slower than actin flow. The difference, however, was only significant in the cell center possibly indicating a reduced degree of coupling (Fig. 8D,E). Notably, the difference between keratin and actin flow was less at higher migration speeds (Fig. 8B).

## Discussion

The use of image analysis tools allowed us to quantitatively assess cytoskeletal network dynamics in time-lapse fluorescence recordings of migrating nHEKs producing fluorescent reporters. The applied methodology overcame challenges associated with moving cells and the heterogeneities encountered at the single cell level by projecting speed vectors obtained in multiple cells onto an idealized stationary D-shaped cell. The resulting precision and accuracy superseded visual inspection and other currently available analysis methods (FRAP, photoactivation).

In this way, we found distinct patterns of keratin dynamics in spontaneously migrating nHEKs. The overall spatial distribution resembled an adaptation of the flow distribution reported for circular shaped sessile cells^14,33–35^ to the polarized D-shape of migrating cells. Thus, retrograde flow was detected in the entire cell except for the back of the cell where anterograde flow occurred, which, however, is also inward directed. Highest flow was found in the cell periphery, lowest flow in the cell center. Additionally, we observed that keratin flow was dependent on the cell trajectory. Higher keratin flow correlated with higher directionality ratios (Fig. 3). Remarkably, turning of cells was associated with a symmetry break of the subcellular flow pattern resulting in asymmetric keratin flow in the lateral back part of turning cells (Fig. 4). A similar symmetry break was also observed for nHEKs moving on sinusoidal stripes (Fig. 4). We suggest that the observed alterations in keratin flow not only reflect alterations in polarization of migrating cells but also contribute actively to local mechanophysical properties. Thus, increased keratin cycling occurs in regions, where network expansion takes place, i.e. at the leading front and at the convex side of turning cells.

In standard culture conditions, i.e. for nHEKs moving on fibronectin-coated glass, higher keratin flow was shown to be associated with higher migration speed (Fig. 2). Changing the mechanical environment altered migration speed and keratin cycling. In accordance with observations in other cell types migration speed was reduced by increasing ECM coating density (Fig. 6)^1,23^ and nHEKs migrated slower on stiff than on soft substrates (Fig. 7)^24^. Of note, migration speed and keratin flow changed concordantly. As a result, a given migration speed was always associated with a specific speed of keratin flow independent of the mechanophysical environment indicating that both processes are under control of the same mechanisms.

The situation for nHEKs moving on narrow 15 µm-wide fibronectin stripes, however, was more complex (Fig. 5). In addition to the expected reduction of both speed of migration and keratin flow the keratin flow was disproportionally slower for a given migration speed than in all other conditions tested. This observation implies that pseudo-confinement imposes additional, migration-independent restrictions on the dynamics of the keratin system involving additional pathways.

The increased focal adhesion density detected on stiff substrates implicated the actin system as an independent upstream regulator of cell migration speed, since previous studies had shown that changes in substrate stiffness and also in coating density induce changes in actin flow of nHEKs^30^ and subsequently in migration speed^36^. This notion was further supported by correlating actin and keratin flow patterns, whereby actin flow was reduced in areas containing keratin filaments compared to areas without keratin filaments. Furthermore, actin flow appeared to be slightly faster than keratin flow in the different cellular overlap regions. The difference between both decreased with increased migration speed. Taken together, these results support the notion that actin is an upstream regulator of keratin dynamics.

The recent study by Wang *et al*.^37^ adds another layer of complexity by showing keratin isotype-specific effects on keratinocyte migration. They found that loss of K6 increases migration speed and directionality of primary keratinocytes. They further linked this to an increased rate of focal adhesion disassembly and showed that K6-myosin association may regulate the migratory phenotype. The altered focal adhesion dynamics together with reduced and mislocalized desmoplakin were presented as indications for intricate crosstalk between the extracellular matrix, neighbouring cells and the keratin cytoskeleton in collective epithelial cell migration. A remaining conundrum is the observed induction of K6 and K16 upon wounding, which is presumably associated with increased migration. The isotype-specific effects of overexpressing and downregulating specific keratins still remains to be untangled to understand the sometimes opposing results in different contexts (recent review^8^; for effects in keratinocytes see also^38,39^). Another recent study by De Pascalis *et al*.^40^ also linked intermediate filaments to collective cell migration by showing that the intermediate filament network of astrocytes that is composed of vimentin, glial fibrillary protein and nestin controls force distribution through plectin-mediated interaction with the acto-myosin network. The authors further demonstrated that intermediate filaments affect focal adhesion organization and their mechanical coupling to the acto-myosin system.

## Conclusion

We conclude that keratin flow patterns very closely reflect migratory behaviour and are controlled by actin dynamics. Increased keratin flow correlates with increased migration in multiple paradigms. On the other hand, keratin acts as a brake on the actin cytoskeleton presumably responding to pro-migratory cues with a slight delay and by providing a counterbalance to the propulsive actomyosin contractility (for vimentin see^41^). The latter function is supported by the separate anchorage of the actin and keratin system to focal adhesions and hemidesmosomes^29,42–44^, respectively, and the localization of both systems in separate layers with only limited connectivity (this work; cf. see also^8,45,46^). The consequence for mechanical coupling of the two biophysically very different systems is an increase in migratory persistence.

Supplementary Fig. S9 schematically depicts major conclusions of the present study. Mechanical properties of the environment affect density and distribution of focal adhesions, which act as crucial mechanosensors and stabilizers of actin flow. Increased actin flow in turn leads to increased migration speed and enhances keratin flow. Conversely, the expanding keratin filament network together with its hemidesmosomal matrix adhesions slows actin flow and thereby reduces overall migration speed. The slower turnover and slightly slower flow of keratin filaments in comparison to actin filaments may direct and harmonize actin dynamics. The keratin filament cytoskeleton may thus serve as a template for actin network organization and thereby enhance persistence of cell migration. Similar mechanisms have been suggested for vimentin-microtubule interactions in migrating retinal pigment epithelial cells^47^. Persistence of cell migration is crucial for efficient migration in complex 3D environments.

## Material and Methods

### Cell culture conditions

Normal human epidermal keratinocytes (nHEKs) derived from neonatal foreskin were purchased from Cell Systems and handled as previously described^48^. Briefly, nHEKs were grown in Dermalife K Medium Complete without TGFα (Cell Systems) in the presence of penicillin-streptomycin (100 µg.mL^−1^). Cells were passaged using Trypkit (Cell Systems) for trypsinization and were frozen at different passages in Cryo-SFM freezing medium (Promocell). nHEKs were routinely used for experiments at passage P3 corresponding to approximatively 10 population doublings. Cells were seeded at a density of 5 000 cells per cm² either on 24 mm diameter high precision glass coverslips (#1.5 from Marienfeld) for high resolution confocal microscopy, on 12 mm diameter coverslips (#1.5 from ThermoScientific) for structured illumination microscopy or on 35 mm diameter glass bottom dishes (MatTek) for live-cell imaging. Surfaces were coated by incubation with either 17 mg.L^−1^ fibronectin solution (VWR; low coating density) or 33 mg.L^−1^ fibronectin (high coating density) for 30 min at 37 °C each in a total volume of 1.5 mL. Microscopic imaging was done 2 days after seeding, when cells were still not confluent (less than 20% confluency).

### Immunoblotting

For extraction of the keratin insoluble fraction, nHEKs were grown in six 100 mm-diameter dishes until they were confluent and processed as previously described^49^. In short, cells were scraped off in 750 μL low-salt buffer (10 mM Tris pH 7.5, 140 mM NaCl, 5 mM EDTA, 2 mM phenylmethanesulfonyl fluoride, protease inhibitor tablet (Roche)) after they had been washed with ice-cold PBS. The cells were homogenized with the help of an Ultra TURRAX T8 mixer (IKA Labortechnik) and the resulting homogenate was centrifuged at 5 000 × g for 10 min at 4 °C. The pellet was resuspended in 1 mL high salt buffer (1% Triton-X 100, 1 mM dithiothreitol, 1.5 M KCl, 2 mM phenylmethanesulfonyl fluoride, protease inhibitor), incubated on ice for 30 min and centrifuged at 15 000 × g for 10 min at 4 °C. Then, the pellet was homogenized again in 1 mL high salt buffer, incubated on ice for 10 min and centrifuged at 15 000 × g for 10 min at 4 °C. Finally, after resuspending the pellet in low salt buffer and distilled water, each followed by centrifugation, the pellet was resuspended in 2x SDS sample buffer (60 mM Tris, 1.7% SDS, 8.3% glycerol, 0.34 M beta-mercaptoethanol, 0.002% bromophenol blue) and stored as the insoluble keratin fraction.

The protein samples were separated by SDS polyacrylamide gel electrophoresis either stained with Coomassie Blue or transferred onto methanol-presoaked polyvinylidene fluoride Immobilon-P membrane (Merck Millipore) by electroblotting in transfer buffer (130 mM NaCl, 50 mM Tris base, 0.1% Tween-20, pH 7.6) at 100 V for 1 h using a Mini Trans-Blot Cell (BioRad). The membrane was blocked using 1x Roti-block (Carl Roth) for 1 hour at room temperature, followed by primary antibody incubation at 4 °C overnight. After three washes with TBS-T (50 mM Tris, 150 mM NaCl, 0.05% Tween 20, pH 7.6), the membrane was incubated with horseradish peroxidase-labelled secondary antibody at room temperature for 1 h, followed by three washes with TBS-T. The antibody detection was done using the Fusion SL chemiluminescence system (Vilber Lourmat) with AceGlow kit (PEQLAB) treated membranes.

The following primary antibodies were purchased: Guinea pig anti keratin 5 (GP-CK5, Progen; 1:5 000) and mouse anti-keratin 17 (Mo-Ab E3; 1:100^50^). All other primary antibodies were kindly provided by Dr. Lutz Langbein: Guinea pig anti keratin 14 (CK 14.2; 1:80 000^51^), guinea pig anti keratin 1 (K1.1; 1:200 000^52^), guinea pig anti keratin 10 (K10.1; 1:1 000^53^), guinea pig anti keratin 6 (K6/2.1; 1:4 000^52^), and guinea pig anti keratin 16 (K16.1; 1:150 000^52^). The following secondary antibodies were used: HRP-coupled rabbit anti guinea pig (P0141, Dako; 1:4 000) and HRP-coupled goat anti mouse (P0447, Dako; 1:2 000).

### Immunofluorescence microscopy

Immunofluorescence labeling was carried out using standard protocols (cf.^48^). Fixation was performed without any washing steps in order to avoid cell retraction. Three different fixation methods were used: (*i*) For methanol acetone fixation, cells were fixed for 2 min in methanol at −20 °C and immediately permeabilized in acetone at −20 °C for 20 s. (*ii*) For paraformaledehyde (PFA)-acetone fixation, cells were first fixed for 10 min at room temperature in 4% (w/v) PFA (Merck) in PBS and were then permeabilized in acetone at −20 °C for 30 s. (*iii*) For PFA-Triton X fixation, cells were first fixed for 10 min at room temperature in 4% (w/v) PFA in PBS, and were then permeabilized in 2% Triton X-100 (Sigma) in PBS solution for 10 min at room temperature.

For staining, fixed cells were passivated for 20 min at room temperature in a solution of 5% (w/v) standard grade bovine serum albumin fraction V (BSA; Serva) in PBS. Incubation with primary antibodies in a solution of 1% (w/v) BSA in PBS was performed for 90 min at room temperature and followed by three washing steps with PBS. Incubation with the secondary antibodies suspended in PBS with 1% BSA was performed for 35 min at room temperature and followed by three washing steps. Mounting reagent was Mowiol (Carl Roth).

A complete list of antibodies used for immunofluorescence is provided in SI Table 1.

### Transfections and constructs

Cells were transiently transfected one day after seeding with transit-keratinocyte transfection reagent according to the protocol provided by the manufacturer (Mirus Bio LLC) as described previously^48^. A total amount of 2.5 µg DNA was mixed with 3.75 µL reagent and 250 µL medium for each 35 mm-diameter dish.

The K5-YFP and LifeAct-RFP constructs have been described^14,54^. The pEYFP-N1 construct was obtained from Clontech (#6006-1).

### Micropatterning

Deep-UV micropatterning was performed according to the protocol described in^48^. Briefly, 30 mm diameter coverslips (Thermo Scientific) were first spin-coated (spin coater KW-4A from Chemat Technology) with TI PRIME (MicroChemicals) and then with polystyrene (Sigma-Aldrich). Afterwards, they were exposed to deep-UV (UVO Cleaner 42–220, Jelight Company Incorporation) for 5 min using the preset conditions. They were subsequently incubated in poly(L-lysine) grafted poly(ethylene glycol) (PLL-g-PEG; SuSoS) and then exposed to deep-UV for 3–8 min with a custom-designed quartz mask (manufactured by Compugraphics) in the same device. Finally, they were incubated in human fibronectin (5 µg.µL^−1^) in 100 mM NaHCO_3_ (pH 8.5). Coverslips were fixed at the bottom of 35 mm-diameter dishes with 18 mm diameter holes (Cell E&G) with GeneFrame tape (ThermoFisher), before cells were seeded at a density of 6 600 cells.cm^−2^.

### Preparation of elastic substrates

Elastic substrates were obtained by spin-coating a silicone elastomer PDMS formulation (Sylgard 184; Dow-Corning) on thin glass coverslips as described^55^ in order to obtain a total thickness that is optimal for imaging, i.e. 170 µm. Mixing different ratios of silicone oil and curing agent allowed the fabrication of substrates with different stiffness. In detail, a mass m_1_ of silicon oil was measured. Then, a mass m_2_ of curing agent equal to m_2_ = α m_1_ with α = 1/10 for a resulting substrate Young modulus of 1.2 MPa, or α = 1/70 for 1.5 kPa, was measured in order to obtain the desired stiffness. The two compounds were manually mixed for 5 min, and degassed for 20 min. Then, the degassed mixture was spin-coated on a glass coverslip (22 mm × 22 mm, #0, Menzel-Gläser) at 1800 rpm for 15 s. For use in live-cell imaging, the coated coverslip was immediately coupled to 35 mm dishes with an 18 mm diameter hole (Cell E&G). Curing was performed for 16 h at 60 °C.

### Imaging conditions

As described previously^48^, structured illumination fluorescence microscopy was performed with an ApoTome.2.microscope (Zeiss) equipped with an oil immersion objective 63x (N.A. 1.4, DIC, Plan apochromat). Live-cell imaging and 3D Z-stacks of immunostained samples were performed using an LSM 710 DUO confocal microscope (Zeiss) equipped with a DefiniteFocus device (Zeiss) and an identical objective. Live-cell imaging was performed in a humidified chamber with 5% CO_2_ set at a temperature of 37 °C.

### Image analysis

#### Manual cell tracking

For tracking of cells based on images acquired over-time with a 20x objective (N. A. 0.8) and a bright field camera. Cell Tracker program^56^ developed in Matlab was used in the manual mode.

#### Quantitative analysis of cytoskeletal dynamics (CMove; Supplementary Fig. S3)

Image sequences acquired with the 63x objective in one or two fluorescent channels were analyzed with a custom designed program developed using Matlab in the following way:

1. Extraction of a cell mask from each frame of the sequence. To this end the fluorescence micrograph was thresholded (average brightness of image as cut-off intensity), holes in the mask were closed by steps of topological opening and closing (disk-shaped structuring element, 3 pixel radius and 10 pixel radius), finally the convex hull was used as cell mask.
2. For determination of trajectories the centroid of the mask was taken as position of the cell.
3. All cell masks were aligned (translation and rotation) by image registration (monomodal).
4. To calculate the flow within the entire mask a square grid was generated (lattice constant 10 pixels) and template regions centered around the grid points (typical template size 31 by 31 pixels) were searched for (search width 10 pixels) by normalized cross-correlation (cut-off 0.6) in the following frame of the sequence.
5. Optional: For sequences with two channels (e.g. actin and keratin) a reference channel was chosen and processed as described above. Alignment of the images of the additional channel was done using the parameters retrieved for the reference channel. However, here flow was calculated within an independent mask that was determined from the additional channel exactly as described in Step 1 (Supplementary Fig. S8).
6. Optional: Normalization of the flow to a standard cell shape. Cells were mapped from the mask of the reference channel to a user defined convex or elongated cell based on the typically encountered cell shape using the algorithm described in^30^. In case of two channels, the additional channel was mapped by the scaling factors determined before for the reference channel. If the mask in the additional channel was larger than that for the reference channel, this resulted in flow values outside the standard shape of the cell defined by the keratin cytoskeleton (Supplementary Figs. S5, 8).

#### Standard image manipulation

General procedures including adjusting contrast, merging channels, performing z-projections, plotting kymographs, etc. were performed using Fiji.

#### Cell eccentricity

Cell eccentricity was defined in Matlab by fitting the cell mask to an ellipse and then determining the ratio of the short axis size of the cell to the long axis size.

### Characterization of trajectories and cytoskeletal flow

Cell trajectories were constructed from the centroids of the cell masks determined by the CMove program (see previous paragraph). Their positions in the reference frame of the laboratory (or dish) are given by the vectors ${\overrightarrow{R}}_{i}$. The instantaneous velocity of a cell during the *i*th step is ${\overrightarrow{v}}_{i}=\,\frac{\Delta {\overrightarrow{R}}_{i}}{\Delta t}$ where $\Delta t$ denotes the frame interval. The average speed of a trajectory consisting of N steps (or N + 1 positions) is $\langle v\rangle =\frac{1}{N\Delta t}\mathop{\sum }\limits_{i=0}^{N-1}\Vert \Delta {\overrightarrow{R}}_{i}\Vert$ and its straightness was characterized by the directionality ratio DR defined as $DR=\frac{\Vert {\overrightarrow{R}}_{N}-{\overrightarrow{R}}_{o}\Vert }{{\sum }_{i=0}^{N-1}\Vert \Delta {\overrightarrow{R}}_{i}\Vert }$.

Cytoskeletal flow speeds were calculated in the reference frame of the moving cell, that is, after image registration, see step 3 of the CMove algorithm above. In these aligned cell images template regions positioned at $\overrightarrow{r}$ in the cell frame in image i were searched for in image i + 1, see step 4 of the CMove algorithm above. This resulted in displacement vector fields ${\overrightarrow{u}}_{i}(\overrightarrow{r})$. Mean cytoskeletal flow speeds were calculated by averaging these vector fields over the whole analysis area and the whole duration of the trajectory. In the case of shape normalization the vector fields were transformed as described in^30^.

### Statistical analysis

All statistical analyses were performed with GraphPad Prism software. For every graph, mean ± SD are plotted, except for Supplementary Fig. S7 where the 5–95% confidence intervals are plotted. Distributions were considered Gaussian if they passed the d’Agostino & Pearson k2 test with a non-significant P value. If both distributions were Gaussian, testing was performed with an unpaired Student t-test for comparison of two conditions. If variances turned out to be significantly different, Welch’s correction was added. When at least one of the distributions was not Gaussian, a Mann-Whitney test was used. When at least three conditions were compared, one-way analysis of variance (ANOVA) followed by Tukey’s test was used. If all distributions were Gaussian, Kruskal-Wallis test followed by Dunn’s test was used on all selected pairs of columns in the reverse case. For correlation analyses, Pearson test was used for Gaussian populations, Spearman test when otherwise. In case of positive results, both were followed by linear regression. *Shows a P-value with P < 0.05, ** for P < 0.01, and *** for P < 0.001. n.s. means non-significant.

## Supplementary information

Supplementary Information.

Supplementary Movie 1.

Supplementary Movie 2.

Supplementary Movie 3.

Supplementary Movie 4.
