# Supplementary material for: Regulation of keratin network dynamics by the mechanical properties of the environment in migrating cells
Source: Sci Rep. 2020 Mar 12;10:4574. doi: 10.1038/s41598-020-61242-5 (PMC7067805; doi:10.1038/s41598-020-61242-5)
Supplement: Supplementary file 1 — Supplementary Information. [file 41598_2020_61242_MOESM1_ESM.pdf]

Supplementary Information

Regulation of keratin network dynamics by the mechanical properties of the environment in migrating cells

Anne PORA[1], Sungjun YOON[1], Georg DREISSEN[2], Bernd HOFFMANN[2], Rudolf MERKEL[2], Reinhard WINDOFFER[1], Rudolf E. LEUBE[1,*]

[1] Institute of Molecular and Cellular Anatomy, RWTH Aachen University, 52074 Aachen, Germany

[2] Institute of Biological Information Processing 2, Forschungszentrum Jülich, 52425 Jülich, Germany

[*] Corresponding author

Rudolf Leube

Institute of Molecular and Cellular Anatomy

RWTH Aachen University

Wendlingweg 2

52074 Aachen, Germany

Phone: ++49 241 80 89107

Fax: ++49 241 80 82508

Email: rleube@ukaachen.de

Supplementary Figures

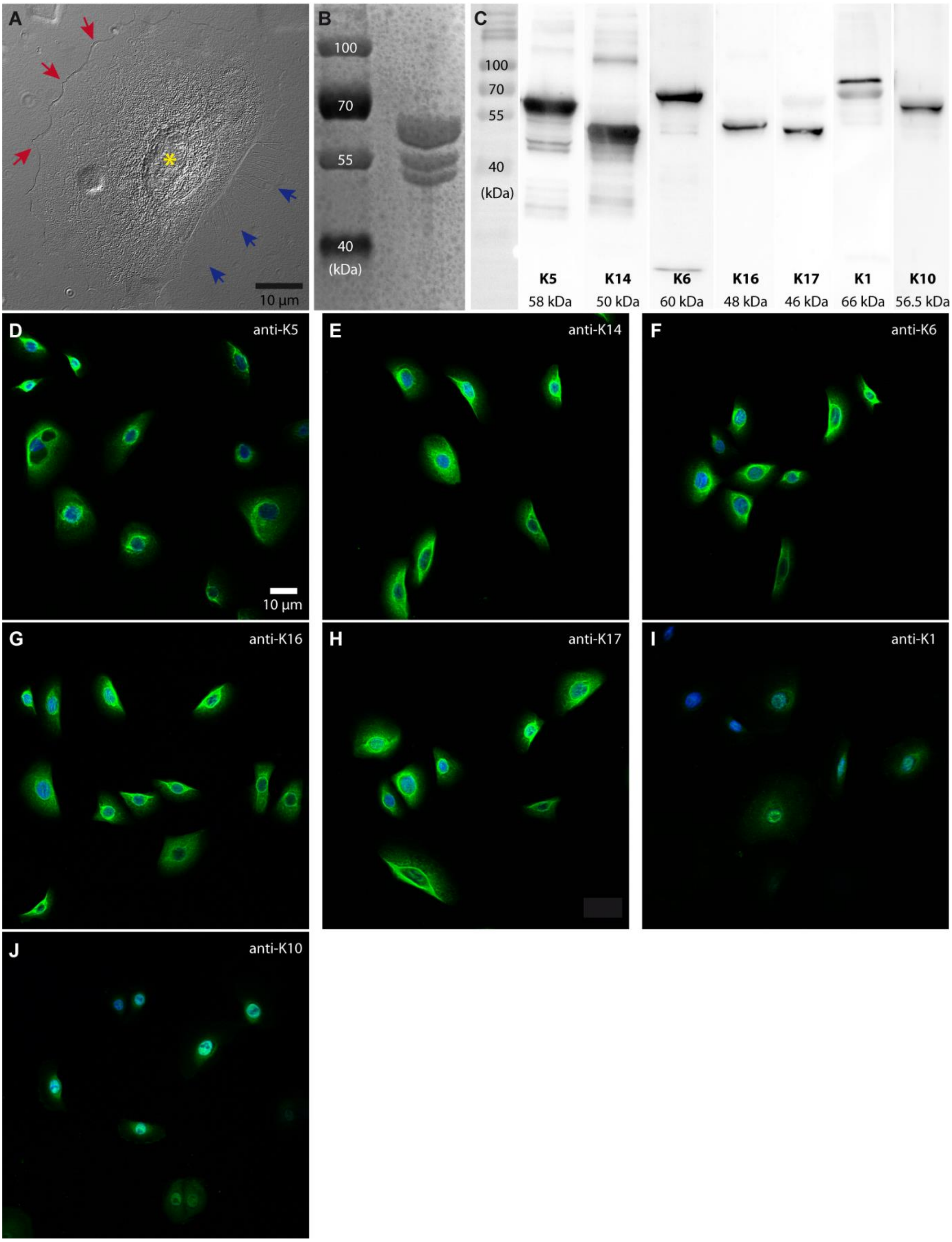

Figures S1. Migrating normal human epidermal keratinocytes (nHEKs) are useful for measuring keratin filament dynamics. (**A**) Live-cell phase contrast image (objective 63 x) of a migrating nHEK. The outlined cell contour reveals a polarized D shape with multiple lamellipodia and filopodia at the curved cell front (red arrows) and prominent retraction fibers at the straight cell rear (blue arrows). The nucleus (yellow asterisk) is shifted toward the back of the cell. (**B**) Coomassie Blue staining and (**C**) complete immunoblots of 10% SDS-polyacrylamide gels after electrophoretic separation of high salt buffer extracts prepared from nHEKs using antibodies against K5 (3 s exposure), K14 (1 s exposure), K6 (1 s exposure), K16 (0.5 s exposure), K17 (60 s exposure), K1 (90 s exposure), and K10 (15 s exposure). The co-electrophoresed size markers are shown at left. (**D-J**) Immunofluorescence images detecting specific keratins. Figure section C is modified from[1].

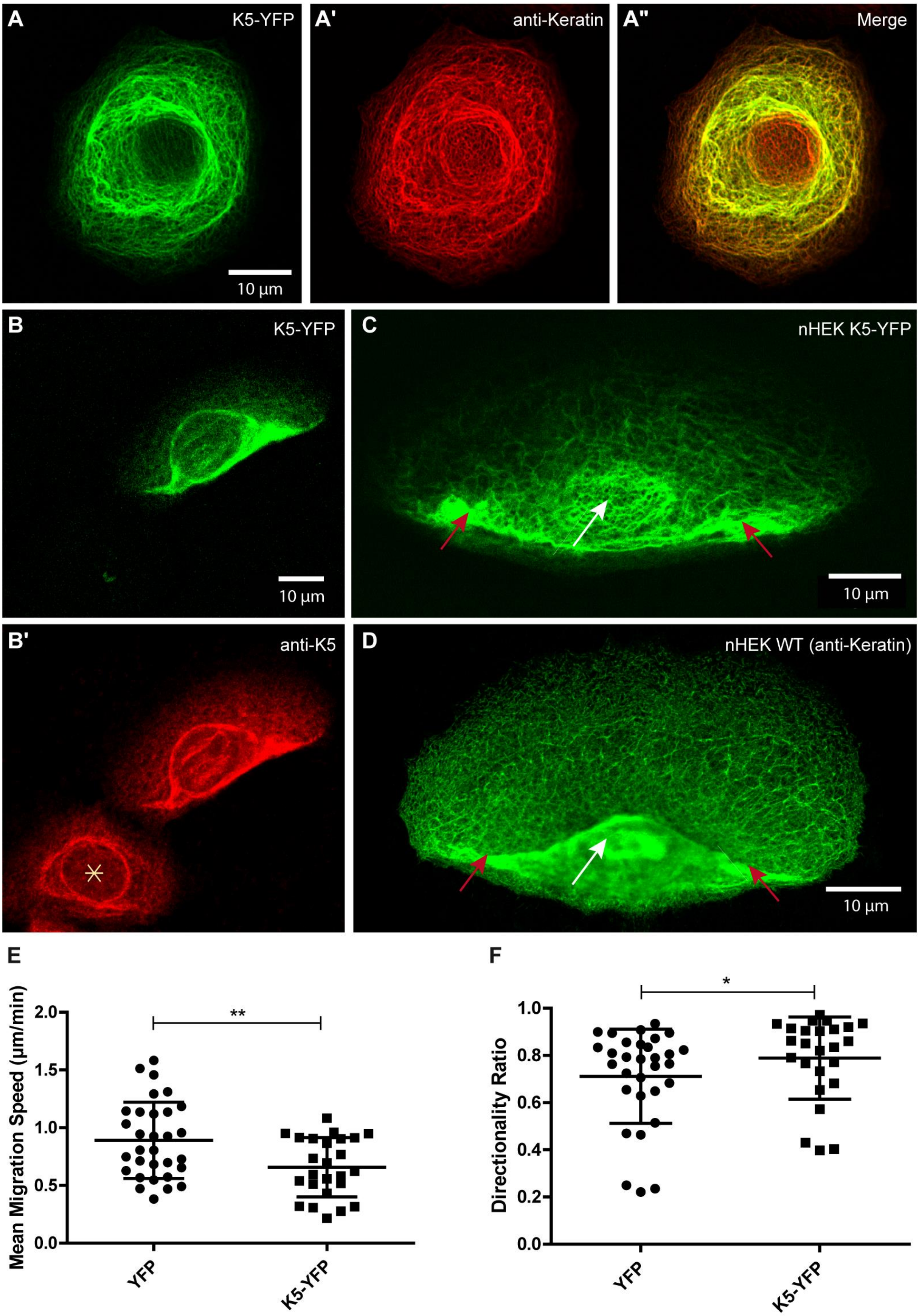

Figure S2. Normal human keratinocytes (nHEKs) are a useful system to monitor fluorescent keratin reporter dynamics. (**A**) Confocal fluorescence microscopy (maximum intensity projections, objective 63 x) of a methanol-acetone fixed non-migratory nHEK producing K5-

YFP after immunostaining with murine pan-keratin antibody cocktail PAN-CK. Note the perfect overlap of the fluorescent reporter (shown in green in A) with the immunosignal (A'; merged image in A''). (**B, B'**) shows a comparison of the confocal fluorescence image of a migrating nHEK K5-YFP reporter (B) and the corresponding immunofluorescence detecting the transgene together with the endogenous keratin network using a keratin 5 antibody (**B'**). Note the co-localization of both and similarity in network organization in the adjacent non-transfected cell *. (**C, D**) show a comparison of a confocal fluorescence image of a live migrating K5-YFP nHEK (C; taken from Movie 1) with a structured illumination fluorescence image of a migrating nHEK after fixation with methanol-acetone and immunostaining with PAN-CK antibodies (E). Note the similarity in overall keratin distribution with prominent lateral extensions of keratin bundles from the nucleus (red arrows) and a cytoplasmic network thinning towards the cell front. All scale bars, 10 µm. (**E, F**) The column scatter plots depict mean migration speeds (E) and directionality ratios (F) ± SD of control nHEKs synthesizing YFP (n = 31) and nHEKs producing K5-YFP (n = 25). Values were extracted from live-cell confocal images (objective 63 x) recorded every 60 s for 30 min in migrating nHEKs. K5-YFP transfection induces a mild decrease in the migration speed and a mild increase in directionality as compared with YFP transfection. Unpaired Student t-test test in F (P = 0.0053); Mann-Whitney test in G (P = 0.0401). Figure sections A-A'' and E-F are modified from[1].

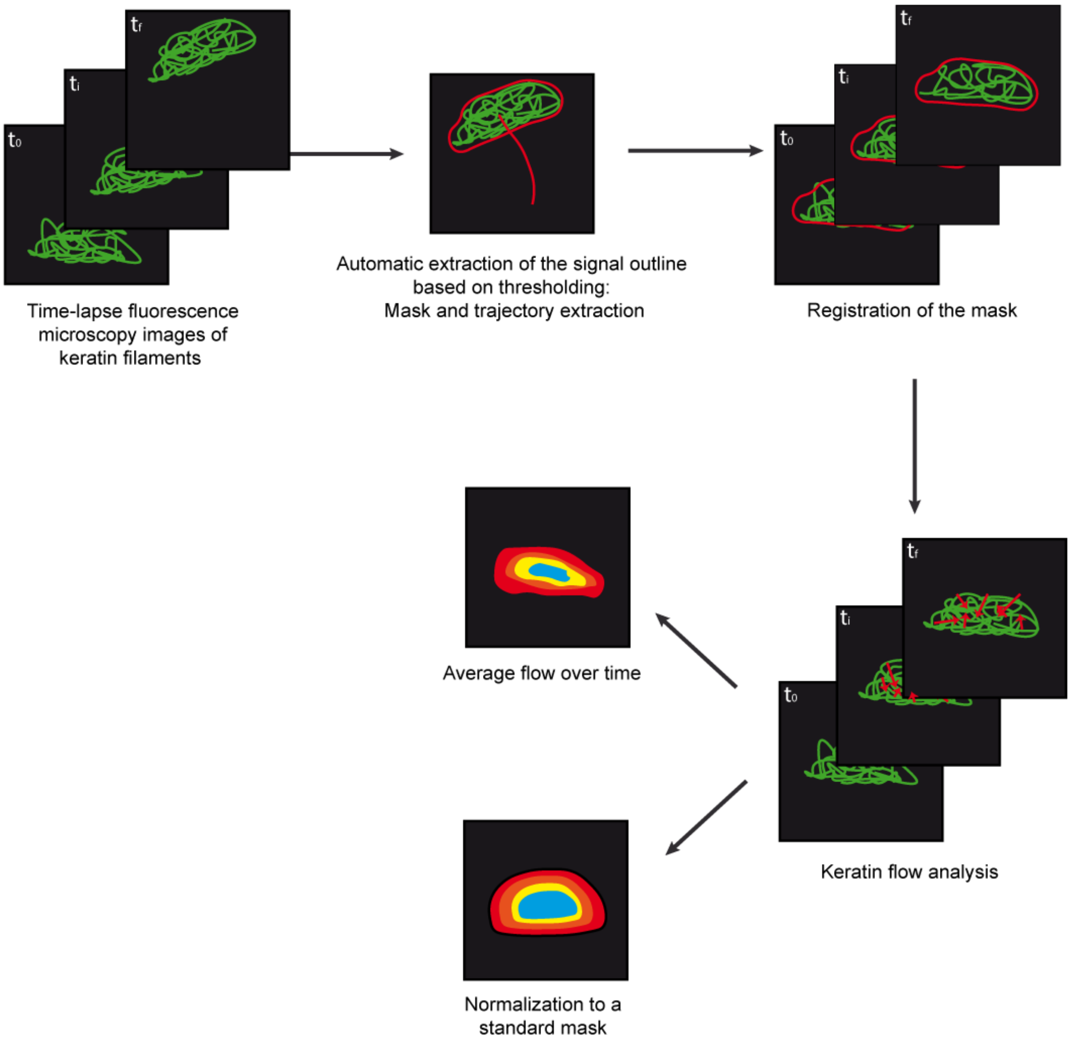

Figure S3. CMove program routine for keratin flow measurements (1 channel)**.** The keratin fluorescent channel is used for analysis. For each frame, the signal outline is extracted automatically by thresholding to create a mask. The centroid of the mask is extracted; cell trajectory and speed are calculated from the sequence of centroid positions. Subsequently, the mask is registered. This corrects for translation and rotation of the cell. In other words, this step is the transformation from a reference frame where the dish is at rest to the cell-based reference frame. Keratin motion analysis is then performed using an algorithm based on cross-correlation. The keratin flow is averaged over time. It is finally normalized to a standard D-shaped mask. The figure is modified from[1].

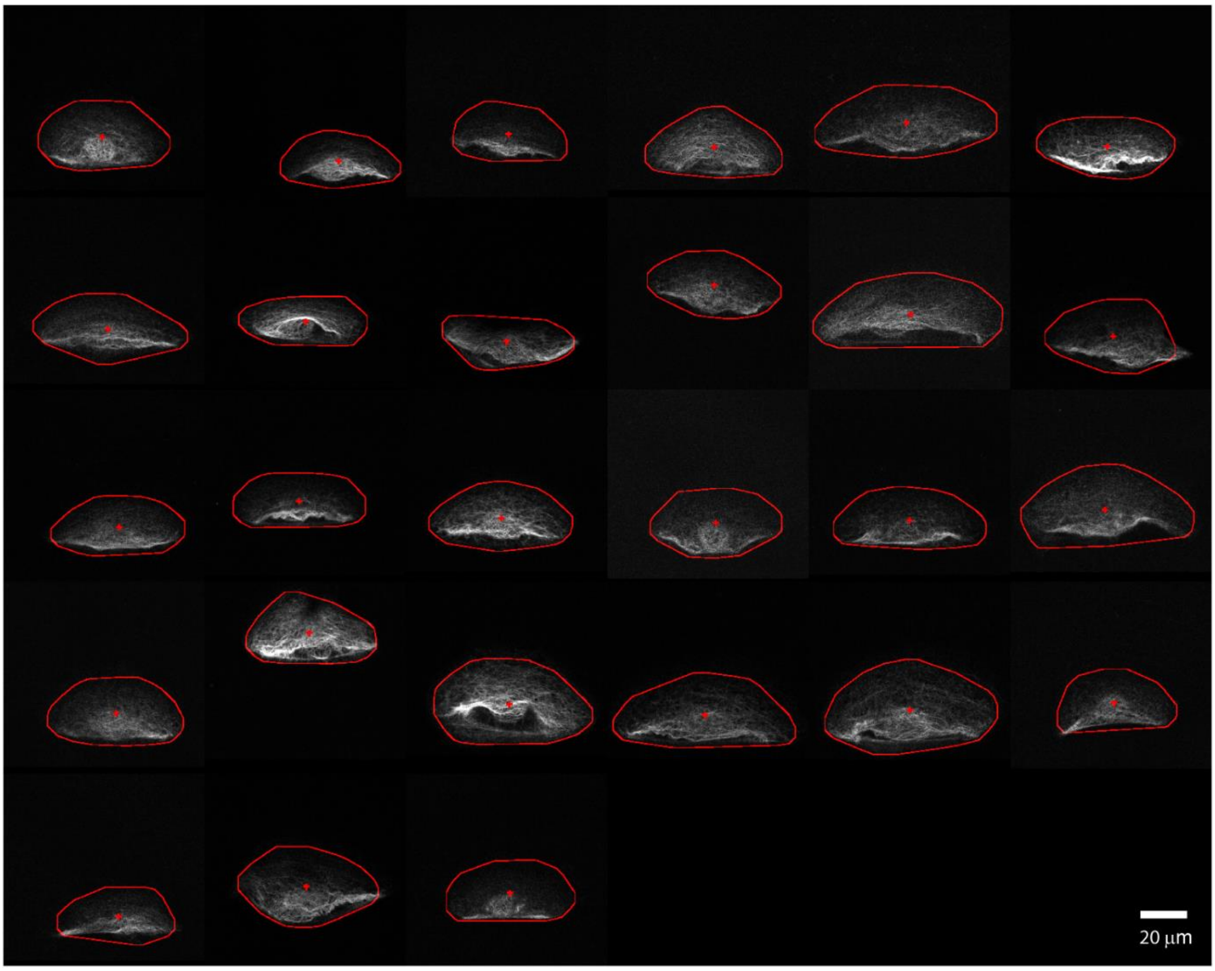

Figure S4. Montage of the fluorescence images recorded at time point 0 of migrating normal human epidermal keratinocytes used for the analysis done in Figs. 1-3. The fluorescence was used as the template to define cell shape, demarcated in red, prior to shape normalization. The center of mass is marked by a cross.

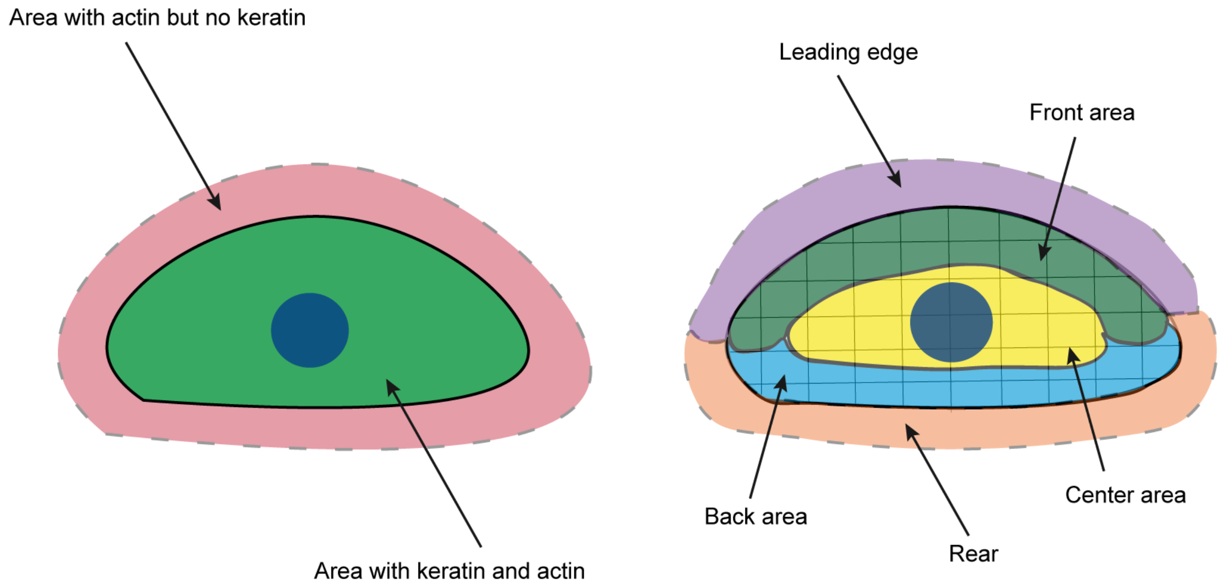

Figure S5. Areas of interest in normalized cells. On the left panel, two different zones are represented: in green the area where both keratin and actin can be detected. This zone contains all the keratin signal. Technically, this area is defined as the cell mask extracted from the keratin signal. We give the green area a standard shape upon normalization of the flow. The region where some actin can be found but no keratin is depicted in pink. It has a variable shape, even after normalization of the keratin flow to a standard shape. We are interested in the green area only in cells where both actin and keratin flow are calculated in parallel. The green zone is further divided into three areas of interest: the cell front comprising the lamellipodium (excluding the very front of it where no keratin is detected), the cell center comprising the area behind the lamellipodium and below the nucleus and the back comprising the back of the cell with thick keratin bundles (excluding the very end of it where no keratin is detected). The pink zone is divided into 2 areas: the leading edge comprising the entire front part of the cell where no keratin is detected, and the rear comprising the entire back of the cell where no keratin is detected. The figure is modified from[1].

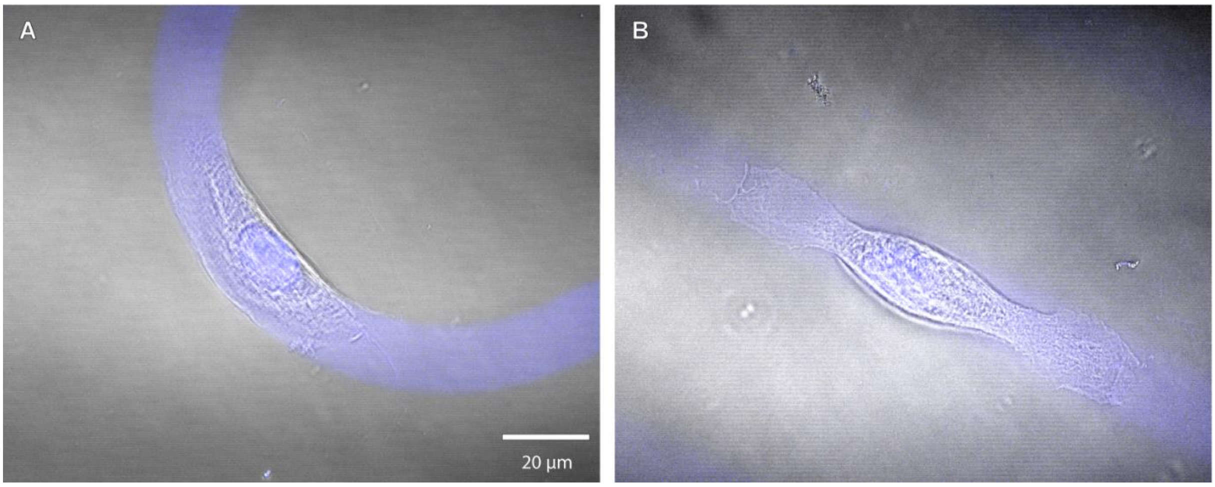

Figure S6. Interference contrast images of normal human epidermal keratinocytes migrating on sinusoidal stripes (A) and straight stripes (B). The fibronectin-coated stripes are shown in light blue.

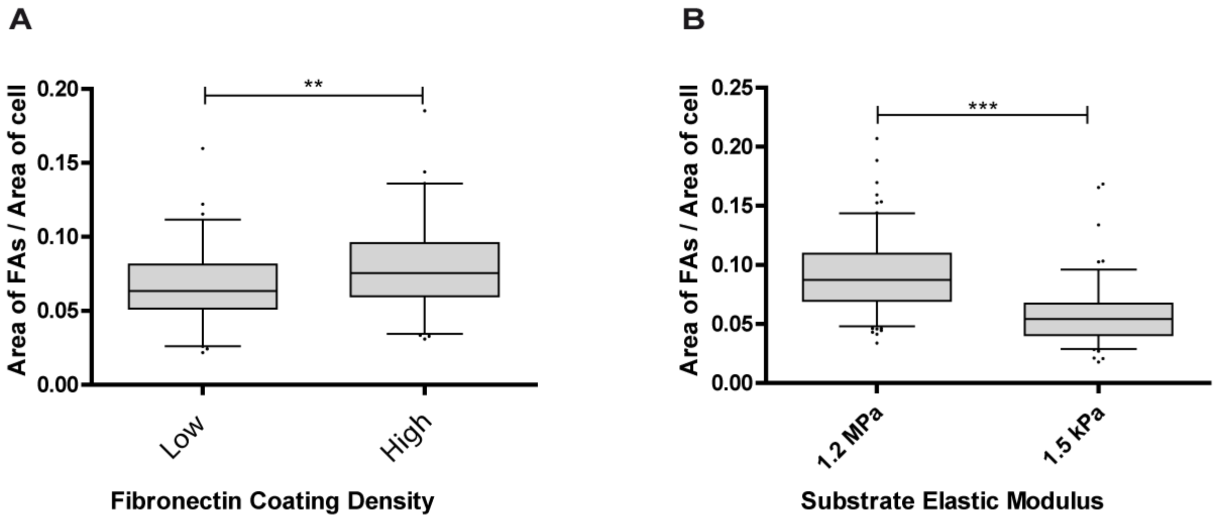

Figure S7. Focal adhesion density scales with fibronectin coating density and substrate elasticity. Quantitation of focal adhesion immunostaining was done by using paxillin antibodies in PFA-acetone fixed normal human epidermal keratinocytes grown on fibronectin-coated glass coverslips (low coating density, n = 75 cells; high coating density, n = 61 cells). The area covered by focal adhesions was determined after manual thresholding of the fluorescence signal recorded by structured illumination microscopy. The total cell area was measured following manual extraction of the cell contour. (**A**) The whisker box plot shows the relative area covered by focal adhesions. Mann-Whitney test was used for statistical analysis ($P < 0.0001$). (**B**) Whisker box plot showing the relative focal adhesion density in nHEKs grown on elastomeric substrates with different stiffness (1.2 MPa, n = 148; 1.5 kPa, n = 114) immunostained for paxillin (PFA-Triton X 100 fixation). Mann-Whitney test was used for statistical analysis ($P < 0.0001$). For cells seeded on softer substrates, the area covered by focal adhesions is lower. The figure is modified from[1].

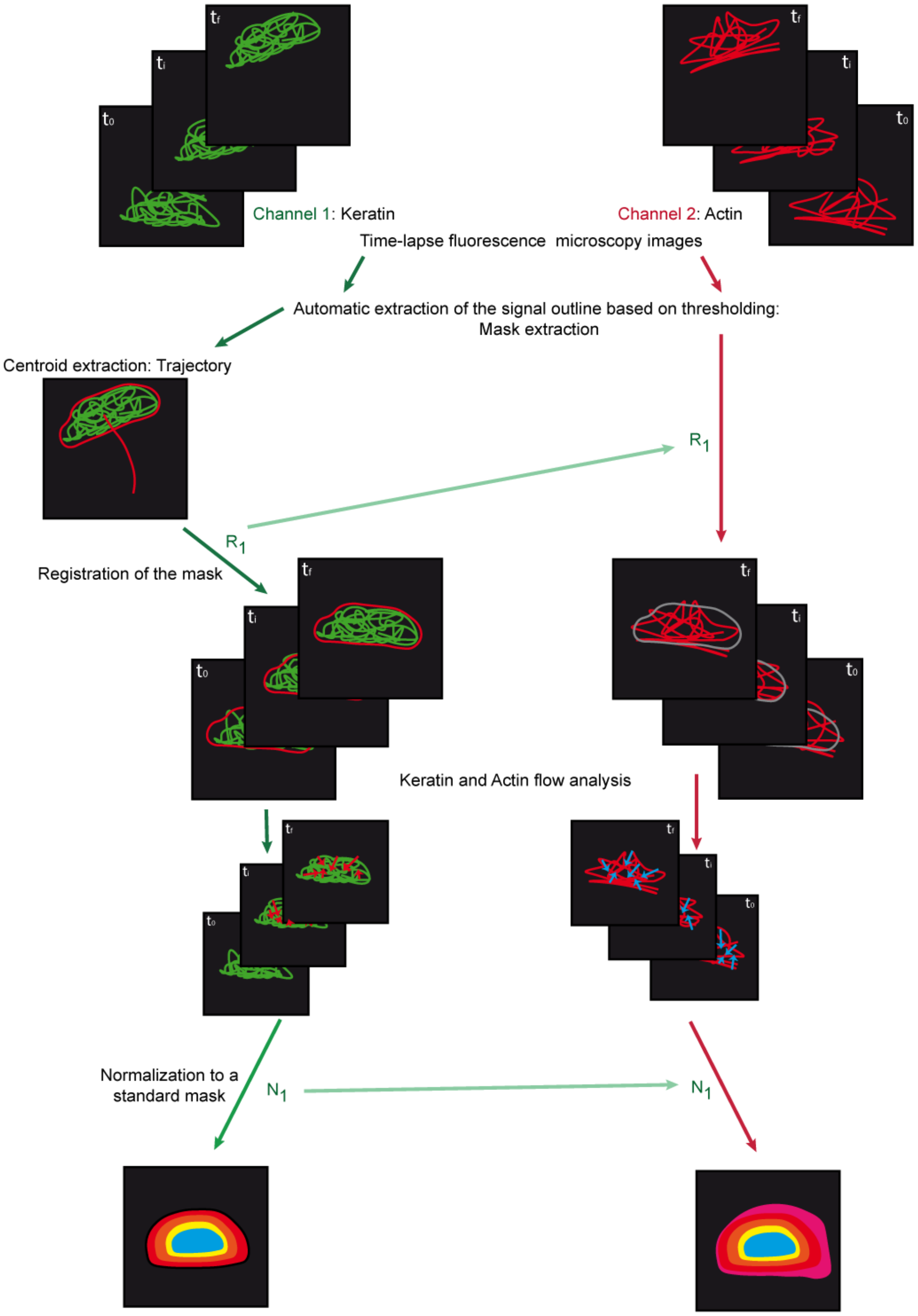

Figure S8. CMove programme routine for parallel measurements of keratin and actin flow. The keratin and actin fluorescent channels are used for analysis. For the keratin channel, the procedure summarized in Fig. S3 is used. For the actin channel, a different mask is calculated that fits the contour of the actin signal. However, it is registered based on the transformation applied to the keratin channel for registration (R_1) (and not based on the movements of the centroid of the actin mask). The actin flow analysis is then performed based on the same

algorithm. It is normalized based on the transformation applied to the keratin channel (N_1). As a result, the actin flow has a different shape for each cell, but the border between the area with keratin and actin and the area with actin only retains the typical D shape. The figure is modified from[1].

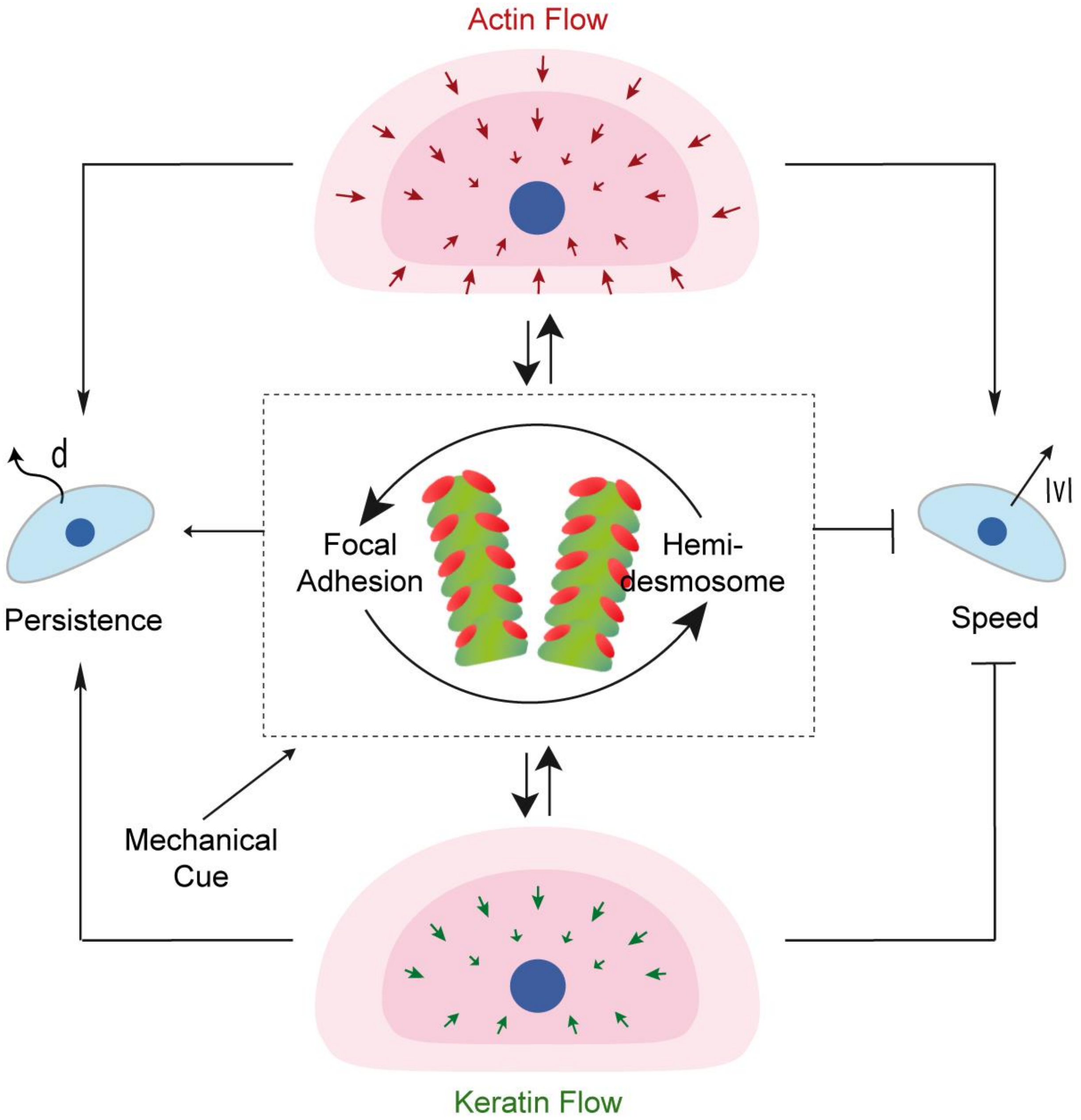

Figure S9. Schematic summary of the co-regulation of actin and keratin flow in migrating normal human epidermal keratinocytes. Mechanical cues elicit changes in focal adhesion (red) distribution, which in turn recruits hemidesmosomal adhesions (green) and alters actin flow. Increased actin flow induces faster migration. Hemidesmosomes together with attached keratin filaments reduce actin flow but also direct it. This results in a decreased speed of migration while enhancing directed cell migration (persistence).

List of Movies

Movie 1: K5-YFP nHEKs migrating on fibronectin-coated glass (corresponding Supplementary Figure S2C)

Laser confocal microscopy images (objective 63x, imaging frequency 1 image.min^{-1}) of a K5-YFP nHEK migrating on fibronectin-coated glass for 30 min. The bright field and corresponding green fluorescent channel are shown. The movie is taken from[1].

Movie 2: K5-YFP nHEK migrating on a fibronectin-coated sinusoidal stripe (corresponding Figure 4B)

Laser confocal microscopy images (objective 63x, imaging frequency 1 image.min^{-1}) of a K5-YFP nHEK migrating on fibronectin-coated sinusoidal stipe for 120 min. The bright field and corresponding green fluorescent channel are shown. The movie is taken from[1].

Movie 3: K5-YFP nHEK migrating on a fibronectin-coated straight stripe (corresponding Figure 5A)

Laser confocal microscopy images (objective 63x, imaging frequency 1 image.min^{-1}) of a K5-YFP nHEK migrating on a fibronectin-coated 15 µm-wide straight stripe for 30 min. The bright field and corresponding green fluorescent channel are shown. The movie is taken from[1].

Movie 4: K5-YFP and LifeAct RFP dynamics in a nHEK migrating on fibronectin-coated glass

Laser confocal microscopy images (objective 63x, imaging frequency 2 images.min^{-1}) of superimposed K5-YFP and LifeAct RFP fluorescence. The movie is taken from[1].

Supplementary Table 1. Antibodies and dyes used for microscopy

Primary Antibodies

Protein Target	Antibody Name	Manufacturer / Catalogue Number / Name of Individual Providing Antibody	Species Raised	Dilution Used
Pan keratin	PAN-CK	ThermoFisher MA5-13203	Mouse	1:200
Paxillin	Clone 349	BD Biosciences, 610051	Mouse	1:100
Keratin 5	Anti-Keratin K5	Progen, GP-CK5	Guinea Pig	1:1 000
Keratin 14	CK 14.2	Lutz Langbein DKFZ	Guinea Pig	1:2 000
Keratin 6	K6/2.1	Lutz Langbein DKFZ	Guinea Pig	1:400
Keratin 16	K16.1	Lutz Langbein DKFZ	Guinea Pig	1:400
Keratin 17	Anti-Keratin 17	Cell Signaling Tech., 4543S	Rabbit	1:500
Keratin 1	K1.1	Lutz Langbein DKFZ	Guinea Pig	1:4 000
Keratin 10	K10.1	Lutz Langbein DKFZ	Guinea Pig	1:100

Secondary Antibodies

Antibody Name	Manufacturer / Catalogue Number	Dilution Used
Alexa-633 goat anti-mouse IgG (H+L)	Invitrogen, A-21053	1:500
Alexa-488 goat anti-mouse IgG (H+L)	Invitrogen, A-11029	1:1 000
Alexa-488 goat anti-guinea pig IgG (H+L)	Invitrogen, A-11073	1:1 000
Alexa-488 goat anti-rabbit IgG (H+L)	Invitrogen, A-11070	1:1 000

Additional Dyes

Dye Name	Manufacturer / Catalogue Number	Dilution Used
Alexa-Fluor-488 phalloidin	Invitrogen, A12379	1:100
DAPI	-	$2ng.\mu L^{-1}$

References

1 Pora, A. *Impact of keratin network regulation on migrating cells* Dr. rer. nat thesis, RWTH Aachen University.1-130. (2019).
